# Quality-Related Monitoring and Grading of Granulated Products by Weibull-Distribution Modeling of Visual Images with Semi-Supervised Learning

**DOI:** 10.3390/s16070998

**Published:** 2016-06-29

**Authors:** Jinping Liu, Zhaohui Tang, Pengfei Xu, Wenzhong Liu, Jin Zhang, Jianyong Zhu

**Affiliations:** 1 College of Mathematics and Computer Science, Hunan Normal University, Changsha 410081, China; pengfei_xu_1@hotmail.com; 2 School of Information Science and Engineering, Central South University, Changsha 410083, China; zhtang@csu.edu.cn (Z.T.); zhang_jin@csu.edu.cn (J.Z.); 3 School of Automation, Huazhong University of Science and Technology, Wuhan 430074, China; lwz7410@hust.edu.cn; 4 School of Electrical and Electronic Engineering, East China Jiaotong University, Nanchang 330013, China; jianyong-zhu@mail.csu.edu.cn

**Keywords:** online product quality inspection, image spatial structure, sequential fragmentation theory, image statistical modeling, Weibull distribution, ensemble learning, semi-supervised learning

## Abstract

The topic of online product quality inspection (OPQI) with smart visual sensors is attracting increasing interest in both the academic and industrial communities on account of the natural connection between the visual appearance of products with their underlying qualities. Visual images captured from granulated products (GPs), e.g., cereal products, fabric textiles, are comprised of a large number of independent particles or stochastically stacking locally homogeneous fragments, whose analysis and understanding remains challenging. A method of image statistical modeling-based OPQI for GP quality grading and monitoring by a Weibull distribution(WD) model with a semi-supervised learning classifier is presented. WD-model parameters (WD-MPs) of GP images’ spatial structures, obtained with omnidirectional Gaussian derivative filtering (OGDF), which were demonstrated theoretically to obey a specific WD model of integral form, were extracted as the visual features. Then, a co-training-style semi-supervised classifier algorithm, named COSC-Boosting, was exploited for semi-supervised GP quality grading, by integrating two independent classifiers with complementary nature in the face of scarce labeled samples. Effectiveness of the proposed OPQI method was verified and compared in the field of automated rice quality grading with commonly-used methods and showed superior performance, which lays a foundation for the quality control of GP on assembly lines.

## 1. Introduction

Product quality is the driving force for every enterprise, which is an important factor to keep an impregnable position in the modern global competitive environment [1,2]. Product quality inspection or monitoring is basically performed by the performance tests of products as well as appearance assessments, to avoid the possible defects in products and ensure customer satisfaction [3,4,5]. The quality of most of types of products can be reflected with their corresponding visual attributes, e.g., glossiness, color, object size, surface coarseness and varieties of defects on the product surface, which are effective sensory indicators for product quality inspection or condition monitoring to a certain extent [6]. Hence, the concept of online product quality inspection (OPQI) with smart visual sensors is attracting increasing interest in both the academic and industrial communities for industrial manufacturing, safety production monitoring, quality control, etc. [7,8,9].

Nowadays, visual sensors-based OPQI is essential and indispensable in most product processing processes, owing to the intrinsic merits of visual inspection technologies, such as fast response, high efficiency, non-intrusiveness, economy, flexibility and so on, particularly on assembly production lines. Recent advances in visual sensor technologies coupled with image processing and analysis technologies have led to flourishing academic studies and engineering applications, in diverse areas such as automotive component manufacture [10], food processing [11], semiconductor production [12], fabric quality inspection [13], nonferrous metallurgy processing [14,15] and many other industrial processes [16,17,18].

There is a kind of special product to be inspected, in quite a lot of applications, namely granulated products (GPs), which are composed of a large number of independent particles or locally homogeneous fragments randomly distributed in the viewing field. Examples are rice, wheat, corn, etc. among food products, greige cloth as a fabric textile, ceramic tile as a building material, and so on. Though the GP image (GPI) is an effective and a direct indicator of the inner quality of the corresponding GP, GPI processing and analysis is not a simple matter.

**Figure 1:**
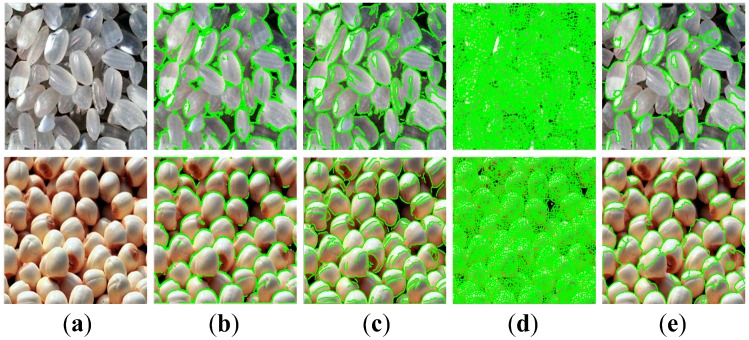
Two GPIs with their image segmentation results by classic image segmentation algorithms. The first line is the rice image, and the second line is the lotus seed image. (**a**) Original GPI; (**b**) image segmentation result by the Sobel operator; (**c**) image segmentation result by the canny operator; (**d**) image segmentation result by the original watershed algorithm [19]; (**e**) image segmentation result by the watershed algorithm integrated with a morphological grayscale reconstruction method [20]. Results from the canny operator, Sobel operator are post-processed by using Otsu’s threshold method.

GPI is a kind of special image without a distinct foreground and background in terms of the morphological structure of GP. It is very difficult to distinguish independent objects (particles or local homogeneous fragmentations) from the GPI based on the commonly-used image segmentation methods. Hence, the physical attributes of individual objects cannot be extracted efficiently and credibly from the GPI, which brings great challenges in GPI processing and analysis and consequently causes a huge obstacle in the OPQI. Figure 1 displays two kinds of typical GPIs with their segmentation results by four different classic algorithms, captured from rice processing and lotus seed screening lines, respectively. As can be seen from Figure 1, commonly-used image analysis technologies are deficient in GPI processing and analysis.

Besides the deficiencies existing in the GPI feature extraction for the OPQI, the classifier establishment is another non-ignorable influencing factor in GP quality identification. Generally speaking, the larger the amount of training samples available for the supervised learning classifier, the better the generalization or performance that can be achieved in a practical application. Unfortunately, the number of the labeled samples is generally small because labeling the samples in practical applications is an expensive and time-consuming task. Only a small quantity of samples can be properly labeled for the classifier training. Most of the samples are unlabeled. Recently, a popular solution in the face of the lack of training samples is the use of semi-supervised learning methods [21], namely, trying to exploit the unlabeled data to help supervised learning.

This study attempts to realize an OPQI system for GP quality grading by introducing a novel GPI feature extraction method based on the spatial distribution of GPI incorporated with a semi-supervised classifier. We introduce a Weibull distribution (WD) model of integral form to do statistical modeling of image spatial structure (ISS) of the GPI. The perceptual significance of the WD-model parameters (WD-MPs), extracted as novel features of the GPI for the following GP quality recognition, was explained based on the theory of sequential fragmentation, which is well known in continued comminution processes. A simple method of weighted summation of a few base filter-responses to gain the responses of omnidirectional Gaussian derivative filter (OGDF), with no requirement for doing the convolution operation at each direction, was introduced to characterize the omnidirectional visual appearance of the ISSs of GPI under various observation scales.

In the classifier construction stage, an ensemble two-classifiers-based semi-supervised learning method was put forward to improve the classification performance of poor results based on the scarcity of labeled samples. Two independent classifiers yet with complementary nature were introduced and trained separately based on the limited labeled samples in advance. Finally, a kind of a co-training-style semi-supervised classifier algorithm for semi-supervised classifier learning was put forward based on the clustering hypothesis in the ensemble learning combining with the parallel characteristic of the bagging multi-learners. The proposed OPQI method was verified on the industrial scale assembly production lines of a food processing enterprise for automated grading of rice quality.

The rest of this paper is organized as follows: Section 2 briefly reviews the related works of GPI processing and semi-supervised classifier learning for OPQI. Section 3 introduces the WD model of the ISS of GPI and makes a thorough analysis of the perceptual significance of the WD-MPs. We address the omnidirectional ISS characterization method by introducing a kind of OGDF in Section 4, and then describe the final ISS statistics features. Section 5 presents the COSC-Boosting algorithm for semi-supervised classifier learning. Section 6 describes in detail the performance of the proposed method in two real case studies, followed by the conclusions in Section 7.

## 2. Related Works

### 2.1. GPI Analysis and Feature Extraction

As can be seen from Figure 1, GPIs are comprised of a large number of locally homogeneous fragments (or particles) in a random arrangement. We can scarcely establish an effective image segmentation method to analyze individual objects in GPIs. GPI processing and analysis remains challenging.

It is worth noting that, the crucial information of GPIs for the OPQI should not be simply attained with a certain fragmentation or a few particles, but should be comprehensive information from the visual appearance of the ISS of the GPI, which is reflected by the spatial distribution, organization or arrangement of the fragments (particles) as well as the shapes of the local visual patterns (fragments) in the observation field. The fragment shape and distribution-dependent visual feature can be essentially attributed to a kind of texture characterization literally [22], ubiquitous in images, but deficient in the definition and difficult to be perceived by computers.

The texture characteristics are inevitably related to the statistical methods. The early widespread methods are the statistics features of images, such as the first order statistics based on some measures, e.g., gray level co-occurrence or difference histograms [23], second order statistics, e.g., Fourier power spectrum [24], gray level co-occurrence matrix (GLCM) [25]; gray level run length matrix (GLRM) [26], and local binary pattern(LBP) [27], multivariate image analysis (MIA) [3], as well as their variants. These methods do not assume any probability model of the ISS, whereas they attempt to extract some first or second statistics as image features in a special transform domain. Unfortunately, the results are possibly misleading or ambiguous in some extreme circumstances. For instance, we can get the same statistics from two sample groups, which actually come from different distributions or distribution models.

As research continued, considerable efforts were devoted to the probabilistic model-based methods to interpret ISS, especially integrated with the prevalent multi-channel image analysis [28], such as Wavelet transform and Gabor filtering. Many useful distribution models are introduced to do statistical modeling of the ISS based on some basic assumptions, e.g., the independent and identically distribution of pixels, and homogeneous spatial assumptions.

In particular, researchers adopted leptokurtosis and fat-tail-shaped distribution models, e.g., Gaussian scale mixture (GSM) [29], generalized Gaussian (GG) [30], Gamma distribution (GD) [31], Gaussian Mixture model (GMM) [32] to characterize the marginal distribution of the image wavelet coefficients, owing to the sparse behavior of the wavelet coefficients. In other words, the marginal distributions of wavelet coefficients are highly kurtotic and long tailed in the coefficient domain or seriously left-skewed in the magnitude coefficient domain. The higher order statistics, e.g., the joint distribution representing the statistical correlations of the pixels in adjustable distances and fixed orientations, is subsequently investigated by augmenting a simple parametric model with a set of hidden random variables that govern the parameters. These established statistical models are used as the prior probability and substantially improve the power of image processing and analysis technologies, such as image denoising [33], foreground or object segmentation [31,32], texture image retrieval [34].

Although model-based methods provide a promising idea for the GPI analysis, current model-based methods conduct limited theoretical analyses of the underlying spatial distribution characteristics of these complex GPIs. These proposed statistical models mainly depend on the experience of experts with the observation of limited image samples. If the predefined statistical models do not really conform to the real distribution profiles of the candidate GPIs, image-based OPQI system may lead to wrong decisions and cause potential economic losses. Thus, the visual appearance with respect to the microheterogeneity, complexity, and uncertainty, and spatial stochastic distribution properties, exhibiting in the GPI remain a great challenge to be depicted effectively [14,35] for the visual sensor-based OPQI.

This work concerns the theoretical statistical distribution of GPIs, introducing an essential statistical model, WD model, by the theory of sequential fragmentation, which is well known in the continued comminution processes. The WD-MPs are then extracted as the GPI feature, whose corresponding significant perceptual meaning is discussed.

### 2.2. Classification

Visual images-based OPQI is essentially a pattern classification or recognition problem. Mathematically, given the labeled example set $L=\left\{\left(x_{L1},y_{L1}\right),\ldots ,\left(x_{Li},y_{Li}\right),\ldots ,\left(x_{LM},y_{LM}\right)\right\},$ the task of OPQI is to assign the proper product quality tag ${\hat{y}}_{t}$ to the probe sample *t* (with the image feature vector *x_t_*), in order to judge whether the product sample *t* is in compliance with the quality requirements.

Many supervised learning methods such as linear discriminant analysis (LDA), support vector machine (SVM), least squares-support vector machine classifier (LS-SVM), linear regression (LR), kernel ridge regression (KRR), artificial neural network (ANN) [36] and their variants can solve this problem. The performance of the existing pattern classification methods mainly depends on the amount of labeled samples as well as their distribution in the whole sample space. Generally speaking, the larger the amount of training samples, the better the performance that can be achieved for every supervised learning classifier. Unfortunately, labeling the samples is expensive in terms of cost and effort in most practical applications. For example, in the OPQI of rice products, rice product grade tags should be assigned based on the aggregative indicators of rice surface gloss, grain size, and the nutritional ingredient assay measured in the laboratory, which is a very tedious and time-consuming work. Hence, although we can easily obtain a great amount of unlabeled rice image samples $U=\left\{\left(x_{U1},-\right),\left(x_{U2},-\right),\ldots ,\left(x_{UN},-\right)\right\}$ by visual sensors, where the strikeout means the corresponding quality label is unknown, only a few labeled samples are available for classifier learning.

Apparently, exploiting unlabeled samples to help supervised classifier learning is a promising solution to solve the scarcity of labeled samples and has been a hot research topic in recent years. To take full advantage of the underlying classification information from the unlabeled samples, semi-supervised learning-based classifier design cause great attention and many successful cases have been reported in the literature, see [37,38,39,40]. Roughly speaking, current semi-supervised learning methods can be categorized into three groups: the first are the generative model-based semi-supervised learning methods. These methods regard the probability of the category labels of the unlabeled samples as a missing parameter, and then the expectation-maximization (EM) algorithm is usually employed to estimate the unknown model parameters [41]. Many commonly-used models are reported in the literature, e.g., Gaussian mixture model [42], and mixture-of-experts system [43]. This method is intuitive and easy to understand and simple to implement, but its accuracy relies on the choice of generative models.

Another are the graph-regularization-framework based methods [44]. These methods usually build a data graph structure based on the marked sample points and unlabeled data points, the tags of the samples are propagated from the labeled points based on the adjacency diagram of the tags to the unlabeled points. Analogously, the performance of these methods also depends on the construction of the data graph.

A third are the co-training methods, which have undergone many improvements [21,45] and have been recognized as one of the main paradigms of semi-supervised learning since they were first proposed [46]. Based on the idea of ensemble learning, more than one, e.g., two, classifiers are established separately on the corresponding sufficient and redundant views. Then, each classifier predicts the labels of the unlabeled samples for the other classifier during the learning process. Predicted labels with high confidence are chosen to augment the training set.

Although co-training methods have been used in many fields, sufficient and redundant views for the corresponding classifiers are required for the traditional semi-supervised learning, which is a condition that cannot be met in many scenarios, especially in practical applications [21,45]. Hence, researchers have attempted to design algorithms that overcome that adverse restriction.

Actually, as stated in [45], with the idea of bagging ensemble learning, different supervised learning classifiers can work without attribute partition or redundant view construction. The labeling confidence can be explicitly measured when a classifier attempts to label the unmarked samples to the other classifier. Hence, researchers have attempted to establish different classifiers by different learning algorithms with complementary prosperities to realize the semi-supervised learning, which do not need the attribute partition and redundant view construction. The appropriate unlabeled samples with high enough confidence labeled by the classifier are chosen to regularize the learning process in order to gain much better generalizationability. More detailed information can be found in [47].

In this paper, a co-training-style semi-supervised classifier named COSC-Boosting algorithm, inspired by the semi-supervised co-training regressor algorithm, COREG [48], is proposed for OPQI. This algorithm employs two different classifiers with complementary natures, each of which labels the unlabeled samples for the other during the learning process on account of the parallel learning property of Bagging ensemble learning. This algorithm does not require sufficient redundant view construction, nor does it require a tenfold cross-validation for label confidence evaluation. These two complementary classifiers are thin plate spline regression classifier (TPSRC) [49] and multivariate adaptive regression spline classifier (MARSC) [50]. TPSRC mines classification information from the overall description of the feature vector of each labeled object, ignoring the local factor interaction in each feature vector (in this paper, any one variable in the GPI feature vector is called a factor). Complementarily, MARSC considers the factor interaction in each feature vector while making full usage of each factor in the feature vector.

## 3. Physical Explanation of the WD model of ISS of GPI

### 3.1. WD Model

As stated in [2,51], any object in the viewing field fragments the scene into two regions, e.g., the internal (foreground or object) and external region (background). In terms of the number of particles in the GPI, the visual scene is inevitably divided into plenty of fragments. The mixture of the shapes, edges, cast shadows with their distributions or organizations of the fragments result in the visual appearance of the ISS of GPI [51].

The best way to describe the ISS of GPI is the spatial statistics of the organization of the texture elements (fragments or particles) using their spatial stochastic aspect. The spatial statistics are the result of an image forming process [51], which is actually equivalent to a single-event fragmentation process of continued comminution in the ore grinding process, which is well studied and can be characterized by the sequential fragmentation theory [2,52].

According to the theory of sequential fragmentation, the probability distribution of the fragmentations of the GPI shows a power-law distribution with the assumption that small details are occurring more often in an image than large structures [53,54]. The resolution power of the visual sensor in real applications cannot be infinite, the fragmentation process of the local particles will inevitably cease. Hence, the statistical distributions of the ISS of the GPI just correspond to the fragments with local contrast larger than a certain fine structure *x* in GPI. Therefore, the statistical distribution model of the ISS of GPI can be described by the integral form WD model [2,51,52]. A detailed demonstration of the WD process of GPI can be found in Appendix A.

The probability density function (PDF) of the WD of integral form is given by a tri-parameter function f(x;μ,λ,β), namely:

(1) $$f\left(x;\mu ,\lambda ,\beta \right)=Ce^{-\frac{1}{\lambda }{\left|\frac{x-\mu }{\beta }\right|}^{\lambda }}$$

where $C=1/\int_{-\infty }^{+\infty }e^{-\frac{1}{\lambda }│\frac{x-\mu }{\beta }{│}^{\lambda }}dx=\;\lambda /\left[2\lambda^{\frac{1}{\lambda }}\beta \Gamma \left(\frac{1}{\lambda }\right)\right]$ is a normalizing constant, only related to the model parameters λ and β and Γ(●) is the gamma function and $\Gamma \left(x\right)=\int_{0}^{\infty }t^{x-1}e^{-t}dt.$

Since we cannot get a closed-form solution of WD-MPs, μ,λ,β, based on the maximum likelihood estimation (MLE), WD-MPs can be estimated by an iterative procedure, the Nelder-Mead simplex algorithm [55], to get the optimal numerical solution. Detailed computation steps can be found in Appendix B.

To evaluate the accuracy of the WD model, the statistics $\chi^{2}$ and Kullback–Leibler divergence (KLD) can be used to measure the goodness of fit, which is computed as follows:

(2) $$\chi^{2}=\sum_{i=1}^{n}\frac{{\left[h\left(x_{i}\right)-f\left(x_{i};\hat{\mu },\hat{v},\hat{\sigma }\right)\right]}^{2}}{f\left(x_{i};\hat{\mu },\hat{v},\hat{\sigma }\right)}$$

(3) $$KLD=\sum_{i=1}^{n}f\left(x_{i};\hat{\mu },\hat{v},\hat{\sigma }\right)\log\left(\frac{f\left(x_{i};\hat{\mu },\hat{v},\hat{\sigma }\right)}{h\left(x_{i}\right)}\right)$$

where *h*(*x_i_*) and $f(x_{i};\hat{\mu ,}\hat{\lambda },\hat{\beta }$) represent the empirical and expected probability on *x_i_*, respectively. Both lower $\chi^{2}$ and KLD values indicate more precise statistical modeling results.

### 3.2. Perceptual Significance of WD Model

The WD model can represent a series of classical statistical distribution by changing the model parameters. For example, when λ = 1, WD becomes the double exponential distribution with a mean value of β. Given λ = 2, WD becomes a Gaussian distribution (GD). Assigning a small value of λ, WD is basically close to the symmetric power-law distribution, given by $f\left(y;\delta ,u\right)=\frac{1}{2\delta }/y-\mu {/}^{-\delta -1},$ where the exponent δ is the measure of fractal dimension [51]. In addition, some studies have shown that the fractal dimension *D_f_* of an image can be estimated by the shape parameter of WD [53], namely, *D_f_* = –3λ.

Researchers have demonstrated that the WD-MPs are directly related to the visual perception characteristics of biological vision systems [51]. With respect to WD-MPs, μ is the location parameter, indicating the global reflectance of the image and it can be derived from the shape-from-shading method [56]. λ is a shape parameter, indicating the grain size with respect to the resolving power, and β is the scale parameter, controlling the width of the distribution. Studies [2,51] have demonstrated that the WD-MPs can make a physical explaination of the human visual perception (HVP) properties [57], such as coarseness, regularity, contrast, roughness, and directionality.

(1) Coarseness or fineness is a fundamental HVP attribute. Commonly, the larger the basis element (fragment or particle) size is, the coarser it is felt in the HVP of the image texture. The coarse texture comes from the magnification of the image accompanied with an increase of resolving power. It can be indicated with the shape parameter λ of the WD model. However, the perception property “coarseness” is evidently related to the observation scale. For example, if we magnify a GPI, but without increasing the resolving power, no new details are included, then the scale invariance will achieved by the HVP with the adaption of the reception field size to the digital magnification of the image. The WD model well fits this kind of scale invariance of the HVP property, namely, a constant shape parameter λ remains. Whereas, if the image magnification with an increase of the resolving power, more details with larger grain are captured in the field of view. Though this process does not affect the WD nature of the ISS, the shape parameter λ becomes smaller in the perception of the “coarse” texture [51].
(2) Regularity is a visual perceptual property regarding the layout variations of the basis texture elements (particles) in the image. In general, a fine texture image tends to be perceived as regular. As addressed above, the coarseness or fineness can be indicated by the shape parameter λ of the WD model. Hence, the shape parameter λ can make a physical explain of the HVP property, regularity. In the extreme case, a few particles or even just one particle exists in the receptive field, namely, the image can be distinguished by a few foreground objects and the remaining background regions. Then, the WD model is rejected, and the spatial distribution of the viewing field can be depicted either by power-law distribution or as pure regular texture (one object fully occupies the entire view of the field). Alternatively, the exhibiting statistical distribution shape is often multimodal when the GPI includes numerous fine particles fully filled in the entire receptive field. This phenomenon can be reflected with the maximum likelihood estimation (MLE) of the shape parameter λ with the resultant of λ≫2 [51], which is the overfitting result of fat tails of the WD model. In this case, the spatial distribution of the grain image is perceived to be regular.
(3) Contrast records the variation range of the illumination intensity or even the color depth of the texture images. The perceptual property “contrast” is essentially caused by the illumination intensity together with the surface height variations of observation objects. The incident and the reflection of the light ray on the object surface codetermine the visual appearance of the ISS with the perception of illumination contrast. The global illumination variation can be reflected by the location parameter μ, which determines the center of the WD and indicates the inhomogeneous illumination surface. Whereas, the surface height variations of the observed objects can be reflected by the scale parameter β, the “width” of WD model, according to the theory of “sequential fragmentation”. Hence, the perceptual contrast can be indicated by two WD-MPs, μ and β [51].
(4) Roughness is originally a tactile property of a surface. Though it seems a 3D property, humans can perceive it with the observation of the 2D image. Roughnes*s* is the perceptual properties results from the height variations of a certain granularity of particles. The greater the height variations of the special sizes of particles are in the view of the field, the rougher perception it is felt. As discussed above, β is an indicator of the height variations of the texture, and the shape parameter λ is the indicator of the granularity of the particles (perceptual coarseness) in the grain image. Hence, perceptual roughness can be reflected by the WD-MPs, β and λ, under a special illumination condition, indicated by the location parameter μ as addressed by Geusebroek [51]. Thus, the combination of these parameters can effectively indicate the roughness of the grain image.
(5) Directionality is a global sense over the entire view of the field. It indicates the dominant orientation of the texture, which is caused by the shapes of the texture elements as well as their placement rules. Though WD-MPs do not include the direct shape information of thetextons(particles), the placement of textons (particles) can be implicitly characterized with WDMPs. Studies [51,53,54] have demonstrated that the anisotropy of grain sizes can be described by the dominant direction of the shape parameter λ. Anisotropy in texture shadows (or contrast) can be reflected by the dominant orientation of the scale parameter β. GPI may exhibit two types of directionalities or anisotropies. The first type is caused by the particle size, and the second type is caused by the contrast variations of particles. Thus, if we fully consider the structural information of the grain image, the HVP-related perceptual attribute, directionality, can be described by the corresponding dominant orientation information of the shape parameter λ and scale parameter β of WD model.

## 4. ISS Characterization and GPI Feature Extraction

### 4.1. Gaussian Derivative Filter (GDF)

Digital images are discrete versions of continuous 2D functions. The image functions at a given point are the finite order truncations of their Taylor series according to Taylor’s theorem. The pixel intensity *I*(*x,y*) at the position (*x,y*) in any local spatial fragment around a predefined original observation point (*x*_0_,*y*_0_) can be determined by the Taylor expansion, namely:

(4) $$\hat{I}\left(x,y\right)=I\left(x_{0},y_{0}\right)\left\{\sum_{n=0}^{K}\frac{1}{n!}\left[\sum_{i=0}^{n}C_{n}^{i}{\left(x-x_{0}\right)}^{i}{\left(y-y_{0}\right)}^{n-i}I_{x^{i}y^{n-i}}\right]+R_{n}\right\}$$

where the term $I_{x^{i}y^{n-i}}$ is a *n*-order mixed derivative of the image function *I* evaluated at the point (*x*_0_,*y*_0_), the orders of partial derivative with respect to *x* direction and *y* direction are *i*, *n – i* respectively; *n*! denotes the factorial of *n*; *R_n_* is the Lagrange residual term.

Equation (4) indicates that the local observed value in the visual pattern is actually determined by the weighted addition of the derivatives at the original observation point over a certain spatial extent (the observation scale), which are derived from the visual appearance ISS. The *n*-order mixed derivatives ($I_{x^{i}y^{n-i}}$) in Equation (4) are closely related to the edge layout, involving the most important spatial structure information of the image *I*. The combination of the mixed derivatives in the Taylor expansion gives a complete representation of the local ISS. $I_{x^{i}y^{n-i}}$ is usually expressed by the GDF, namely:

(5) $$I_{x^{i}y^{n-i}}\left(x,y\right)=\left[\frac{\partial^{i}}{\partial x^{i}}\frac{\partial^{n-i}}{\partial y^{i}}I\left(x,y\right)\right]*G\left(x,y,\sigma \right)=I\left(x,y\right)*G_{x^{i}y^{n-i}}\left(x,y,\sigma \right)$$

where ∗ denotes the convolution operator and $G_{x^{i}y^{n-i}}\left(x,y,\sigma \right)$ is an *i*+ (*n – i*) = *n*-order mixed derivative of Gaussian filters, subject to *i* ≥ 0; *n – i* ≥ 0:

(6) $$G_{x^{i}y^{n-i}}\left(x,y,\sigma \right)=\frac{\partial^{i}}{\partial x^{i}}\frac{\partial^{n-i}}{\partial y^{n-i}}G_{\sigma }\left(x,y\right)$$

$G_{\sigma }\;\left(x,y\right)$ is the Gaussian kernel with the scale parameter σ.

### 4.2. OGDF

To facilitate description, we denote a simple notation $G_{\kappa ,\sigma }$ as a κ-order mixed GDF with scale parameter σ. The use of $G_{\kappa ,\sigma }$ for ISS characterization can reflect the structure information of a GPIat the corresponding *x* coordinate and *y* coordinate direction with respect to the mixed partial derivative of $G_{\kappa ,\sigma }$. As mentioned above, the ISS of GPI is directional. The shapes and directional arrangements of the local homogeneous particles contribute the visual appearance of the ISS of the GPI. In order to take full consideration of the multi-directional ISS, it is necessary to construct the orientated filter for omnidirectional ISS feature characterization.

Intuitively, the response of the image *I* at direction θ results from the convolution operation of the image *I* with the rotated GDF at the corresponding orientation θ. Hence, we should do the convolution operation at all of the directions if we need the omnidirctional ISS features. Unfortunately, this computing style is fairly time-consuming, which is not suitable for GPI processing in the OPQI.

As stated by Freeman [58], the linear combination of several Gaussian derivative filter bases is steerable, hence we can obtain the omnidirectional ISS by linear weighting addition of a limited number of filtering responses of GDFs. Suppose we have the following filter template:

(7) $$G_{\kappa ,\sigma }\left(x,y\right)=\sum_{m=1}^{K}\sum_{i=0}^{m}k_{m,i}\frac{\partial^{i}}{\partial x^{i}}\frac{\partial^{m-i}}{\partial y^{m-i}}G_{\sigma }\left(x,y\right)$$

where $G_{\kappa ,\sigma }\left(x,y\right)$ represents a mixed κ-order GDF with the highest order of κ. As the filter template in Equation (7) is steerable, the filtering response of image *I* with filter template $G_{\kappa ,\sigma }\left(x,y\right)$ at a special rotation angle θ satisfies the following formula [59]:

(8) $$I\left(X\right)*G_{\kappa ,\sigma }\left(XR_{\theta }\right)=\sum_{m=1}^{K}\sum_{i=0}^{m}\alpha_{m,i}I_{m,i}\left(X\right)$$

where **X** = (*x,y*)*^T^*, *R*_θ_ is the rotation factor, $R_{\theta }=\left(\begin{array}{cc} \text{cos}\theta & \text{sin}\theta \\ -\text{sin}\theta & \text{cos}\theta \end{array}\right)$, $G_{\kappa ,\sigma }\left(X,R_{\theta }\right)$ is the rotational version of $G_{\kappa ,\sigma }\left(X\right)$ in the direction of θ, * represents the convolution operation; *I_m,i_*(**X**) is the filtering response of the image *I* with the mixed partial derivative of Gaussian filter $G_{m,i,\sigma }$ with the derivative orders of i and m−i with respect to the *x* and *y* directions, respectively, namely:

(9) $$I_{m,i}\left(X\right)=I\left(X\right)*\underset{G_{m,i,\sigma }}{\underset{︸}{\frac{\partial^{i}}{\partial x^{i}}\frac{\partial^{m-i}}{\partial y^{m-i}}G_{\sigma }\left(x,y\right)}}$$

**Figure 2:**
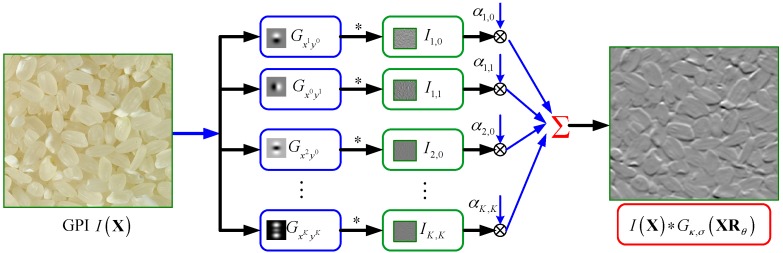
Schematic of weighted summation-based steerable OGDF.

Hence, if we obtain the linear weighting coefficient $\alpha_{m,i}$, we can achieve ISS at any orientation with the steerable filter $G_{\kappa ,\sigma }\left(x,y\right)$ via a weighted summation of several base GDF responses of the GPI *I* at a very low computational cost. The computing mode of the steerable filtering is displayed in Figure 2.

The coefficient $\alpha_{m,i}$ can be computed by using the properties of linearity, rotational and differentiation of the Fourier transform of Equation(8), and the eventual result $\alpha_{m,i}$ is a trigonometric polynomial function of the orientation θ, given by the following computing rule:

(10) $$\alpha_{m,j}=\sum_{i=0}^{m}k_{m,i}\sum_{t=0}^{i}\sum_{l=0}^{m-i}{\left(-1\right)}^{l}C_{i}^{t}C_{m-i}^{l}{\left(\cos\theta \right)}^{t+m-i-l}{\left(\sin\theta \right)}^{i-t+l}$$

The detailed derivation process can be seen in Appendix C.

### 4.3. GPI Feature Extraction

The responses of OGDF always exhibit a periodicity of period π on the orientation map in terms of the properties of steerable GDF. Hence, only the directions in the [0~π] are essential for ISS analysis. We discretize the continuous direction of [0~π] into *N* discrete orientations uniformly for omnidirectional ISS characterization. ISS at the *N* pre-chosen directions (ISSPDs) are computed by linear weighted summation of the responses of the separable GDF bases as expressed in Equation (8). We do statistical modeling of ISSPDs based on the WD model and the WD-MPs are used to generate the feature variables of ISS.

ISS is not only direction-dependent, but also related to the Gaussian scale parameter σ of $G_{\kappa ,\sigma }$. In order to obtain the features of ISS of multi-scales, we employ a series of scales {σ*_i_*│*i* = 1,2,…,*T*} for ISS analysis. Suppose a fixed scale space for ISS analysis is σ, the omnidirectional statistical distribution feature vector, $f_{I,\sigma }^{Global}$ of ISS of a global image *I*, can be expressed as follows:

(11) $$f_{I,\sigma }^{Global}={\left[\mu_{\theta_{1},\sigma },\beta_{\theta_{1},\sigma },\lambda_{\theta_{1},\sigma },\mu_{\theta_{2},\sigma },\beta_{\theta_{2},\sigma },\lambda_{\theta_{N},\sigma },\cdots ,\mu_{\theta_{N},\sigma },\beta_{\theta_{N},\sigma },\lambda_{\theta_{N},\sigma }\right]}^{T}$$

where $\mu_{\theta_{i},\sigma },\beta_{\theta_{i},\sigma },\lambda_{\theta_{i},\sigma }$ represent the WD-MPs of GPI *I* under the Gaussian observation scale σ on thedirection θ*_i_*. Given *T* dimensions of Gaussian scales [σ_1_,σ_2_,…σ*_T_*], the multi-scale omnidirectional statistical feature vector of the ISS of a global image *I* can be characterized as follows:

(12) $$f_{I}^{Global}={\left[{\left(f_{I,\sigma_{1}}^{Global}\right)}^{T},{\left(f_{I,\sigma_{2}}^{Global}\right)}^{T},\cdots ,{\left(f_{I,\sigma_{T}}^{Global}\right)}^{T}\right]}^{T}$$

**Figure 3:**
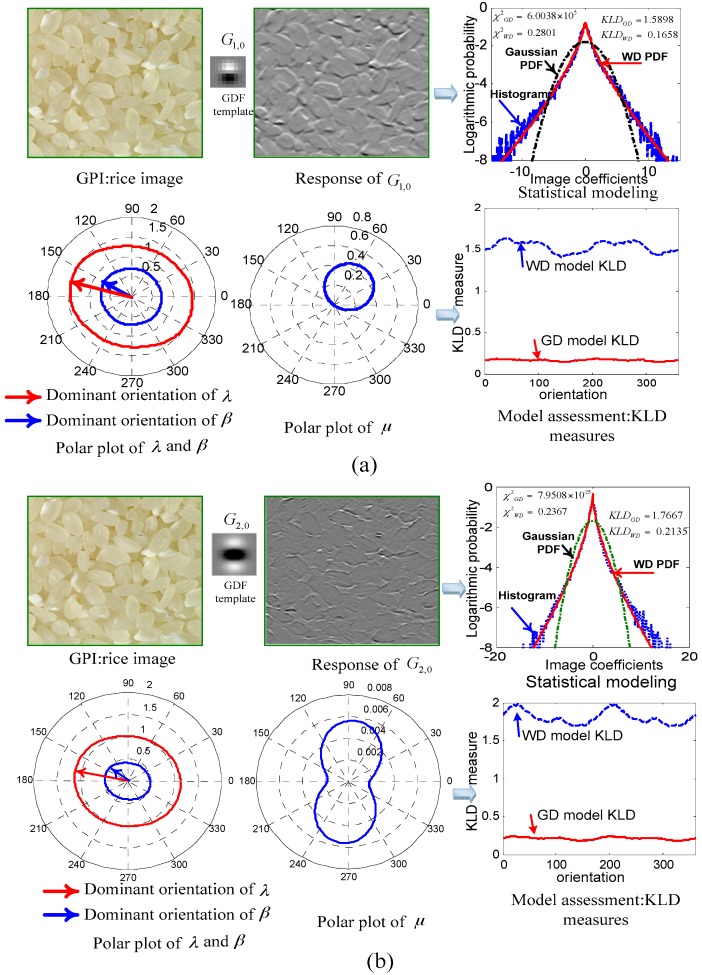
Illustrative example of extracting the omnidirectional ISS feature of a GPI. The omnidirectional WD-MPs are displayed in polar plots with two steerable filter templates with different derivative orders. The fitting accuracies with WD model and GD model ofISS are also compared and displayed with measures of statistics KLD and $\chi^{2}$, which indicate clearly that the WD model is much better than the GD model to do statistical modeling of the ISSI of grain images. (**a**) GPI feature extraction with a first-order GDF template *G*_1,0_; (**b**) GPI feature extraction with a second-order GDF template *G*_2,0_.

Figure 3 displays a rice image with its omnidirectional statistical features of ISS with two different steerable filter templates.

However, the global WD-MPs (GWD-MPs) obtained from the entire filtered images suffer from the loss of the local structure information of ISS. We adopt the analogous processing mode reported in [30] to gain the local information, namely, we split the filtered images into non-overlapping sub-images and each sub-image is treated as an individual image, whose WD-MPs are extracted as the local WD-MPs (LWD-MPs) of GPIs. Finally, GWD-MPs and LWD-MPs are concatenated into an extended ISS feature representation. Finally, the extended WD-MPs feature of a GPI is formulated as:

(13) $$f_{extended,I}={\left[{\left(f_{I}^{GWD-MPs}\right)}^{T},{\left(f_{I}^{LWD-MPs}\right)}^{T}\right]}^{T}$$

where $f_{I}^{GWD-MPs}=\;f_{I}^{Global}$ represents the global feature vector, namely, global WD-MPs of image *I*, $f_{I}^{LWD-MPs}$ means the local feature vector, namely, local WD-MPs of image *I*; $f_{I}^{LWD-MPs}={\left[{\left(f_{subI_{0}}^{GWD-MPs}\right)}^{T},{\left(f_{subI_{1}}^{GWD-MPs}\right)}^{T},\ldots ,{\left(f_{subI_{N-1}}^{GWD-MPs}\right)}^{T}\right]}^{T}$, where *subI_i_* is the *i*-th sub-image in the image *I*, *N* is the number of the non-overlapping subimages sampled in image *I*.

## 5. COSC-Boosting Based Semi-Supervised Learning Classifier

### 5.1. Basic Idea of COSC-Boosting

COSC-Boosting algorithm is a semi-supervised classifier algorithm based on the parallel learning property of Bagging ensemble learning, which employ two classifiers, TPSRC*h_TPSRC_*(**X**) and MARSC *h_MARSC_*(**X**). Each of the classifier labels the unlabeled samples for the other. The predicted labels of the unlabeled samples with high confidence are chosen to augment the labeled data set, to regularize the classifier learning process. The labeling confidence is estimated by consulting the influence of the labeling of the unlabeled samples on the actually labeled samples, namely, the classification error of the classifier on the labeled example set should decrease most quickly if the most confidential unlabeled samples are used [48].

In more detail, COSC-Boosting algorithm repeatedly selects *M*’ unlabeled samples from the unlabeled samples U randomly with replacement. After each selection, the pre-configured TPSRC and MARSC (trained based on the labeled samples) are used separately to pre-label the *M*’ unlabeled samples, and then the samples with high degrees of confidence are applied to the TPSRC and MARSC for classifier model updating and eventually to achieve better classification performance. Such selection of *M*’ unlabeled samples for classifier updating is to ensure the diversity of sample selection on one hand and to avoid the classifier overfitting by restricting the number of potential confident points on the other hand.

In terms of the complementary nature of TPSRC and MARSC, for every unlabeled sample **x***_ui_* in the repeated selection, if TPSRC and MARSRC assign the sample label on **x***_ui_* separately on the current training condition, namely ${\hat{y}}_{ui}=Label\left\{h_{TPSRC}\left(x_{ui}\right)\right\}=Label\left\{h_{MARSC}\left(x_{ui}\right)\right\},$ then the sample $\left(x_{ui},{\hat{y}}_{ui}\right)$ is a candidate high confidence sample. However, only the truly high confidence samples should be used to update the classifier learning. Intuitively, the truly high confidence labeled samples should be the samples that make the classifier consistent with the labeled example set (augment set, including the unlabeled samples but assigned labels with the classifier in the previous learning steps with high confidence level). To evaluate the consistency, the mean square error (MSE) of the classifier on the labeled are used to evaluate the confidence.

The MSE of the classifier utilizing the information provided by $\left(x_{ui},{\hat{y}}_{ui}\right)$ can be evaluated on the labeled example set. Let $h_{TPSRC}^{*}\left(x\right)$ and $h_{MARSC}^{*}\left(x\right)$ be the refined classifiers of $h_{TPSRC}\left(x\right)$ and $h_{MARSC}\left(x\right)$ respectively, which are updated by a high confidential label $\left(x_{u},{\hat{y}}_{u}\right),$ and $\tilde{L}$ be the augmented training sample set. The high confidence labeled examples $\left\{\left(x_{ui},{\hat{y}}_{ui}\right)\right\}$ are identified through the following rule:

(14) $$MSE\left(h_{l};x_{u_{i}}\right)=\sum_{x_{i}\in \tilde{L}}\left\{{\left[y_{i}-h_{l}\left(x_{i}\right)\right]}^{2}-{\left[y_{i}-h_{l}^{*}\left(x_{i}\right)\right]}^{2}\right\}\geq 0$$

where if and only if both $MSE\left(h_{TPSRC}\right)$ and $MSE\left(h_{MARSC}\right)$ satisfy the above conditions at the same time, then the unlabeled sample is confirmed as a high confidence labeled sample candidate and can possibly be used to refine the classifier, in other words, we accept $h_{TPSRC}^{*}\left(x\right)$ and $h_{MARSC}^{*}\left(x\right)$ at the condition when MSE declines and the labeled set is augmented by $\left(x_{u},{\hat{y}}_{u}\right)$ who maximizes the values of $MSE\left(h_{l}\right)$.

### 5.2. TPSRC

Here we take the two-class case as an instance. In this case the class label *y_i_* on the sample feature **x***_i_* belongs to a two-value label set {+1,–1}, e.g., let “−1” represents the “high quality” product and “+1” label the “other quality” product. Given that $L={\left\{x_{Lt},y_{Lt}\right\}}_{t=1}^{M}$ is a training data set consisting of *M* samples, including *M*_1_ sampling points representing the “high quality” product samples, denoted as Ω*^H^*, and *M* – *M*_1_ sampling points of “other quality” product samples, denoted as Ω^0^, *M*_1_ < *M*.

TPSRC is a spline function *h_TPSRC_*. For each point $x_{i}^{H}\in \Omega^{H},\;h_{TPSRC}\left(x_{i}^{H}\right)=y_{i}^{H}\approx -1$ and for each $x_{i}^{0}\in \Omega^{0},\;f\left(x_{i}^{0}\right)=y_{1}^{0}\approx 1.$ This regression or classification task can be solved in a generalized framework with data fitting and function smoothness by solving the following optimal problem, namely, [49,60]:

(15) $$h_{TPSRC}\left(x\right)=\underset{h_{TPSRC}}{\min}\left\{J\left(h_{TPSRC}\right)=\underset{\begin{array}{cc} samples & from\begin{array}{c} \begin{array}{c} \Omega^{H} \end{array} \end{array} \end{array}}{\underset{︸}{\sum_{i=1}^{M_{1}}{\left(-1-h_{TPSRC}\left(x_{i}{}^{H}\right)\right)}^{2}}}+\underset{\begin{array}{cc} samples & from\begin{array}{c} \begin{array}{c} \Omega^{O} \end{array} \end{array} \end{array}}{\underset{︸}{\sum_{i=1}^{N-M_{1}}{\left(1-h_{TPSRC}\left(x_{i}{}^{O}\right)\right)}^{2}}}+\eta s\left(h_{TPSRC}\right)\right\}$$

where *s*(*f*) is the smoothness penalty function on function *f*, and η is the regularization parameter.

Minimizing *J*(*h_TPSRC_*) with different hypotheses will achieve the spline function *h_TPSRC_* of different forms. In this study, we attempt to find the form of function *h_TPSRC_* in the Sobolev space [60], where *s*(*f*) is defined as a semi-norm and researchers have proved that the result of Equation (15) is a unique spline function given by [49,60]:

(16) $$h_{TPSRC}\left(x\right)=\sum_{i=1}^{d}\omega_{i}p_{i}\left(x\right)+\sum_{j=1}^{M}\psi_{j}\phi_{j}\left(x\right)$$

where **x** = [*x*_1_,*x*_2_,…,*xk*] is a *k*-dimensional input vector, φ*_j_*(**x**) is a Green’s function, *d* = (*k + s* – 1)!*k*!(*s* –1)!; *s* is the order of the partial derivative of the semi-norm, satisfying *s* > 0; ${\left\{p_{i}\left(x\right)\right\}}_{i=1}^{d}p_{i}\left(x\right)$ is a set of primitive polynomials, which spans the polynomial space of total degrees less than *s*. The larger the *s* is, the smoother function *h_TPSRC_* will be achieved.

In real applications, researchers usually limit the polynomial space to be linear and consider a kind of radial basis function as the only one form of Green’s function, then the following spline function can be derived [49,60]:

(17) $$h_{TPSRC}\left(x\right)=\omega_{0}+\sum_{i=1}^{k}\omega_{i}x_{i}+\sum_{j=1}^{M_{1}}\psi j^{H}\phi j^{H}\left(x\right)+\sum_{j=1}^{M-M_{1}}\psi j^{O}\phi j^{O}\left(x\right)$$

where $\phi_{j}^{H}\left(x\right)$ and $\phi_{j}^{0}\left(x\right)$ both take the radial basis function as the form of Green’s function, namely, $\phi_{j}^{H}\left(x\right)=│x-x_{j}^{H}{│}^{2}\text{log}\left(│x-x_{j}^{0}│\right)$, $\phi_{j}^{0}\left(x\right)=│x-x_{j}^{0}{│}^{2}\text{log}\left(│x-x_{j}^{0}│\right)$.

In terms of the geometrical meaning of the Equality (17), ω_0_ represents the translation, $\sum_{i=1}^{k}\omega_{i}x_{i}$ reflects the affine transformation, whereas the remaining terms in the Equality (17) record the locally nonlinear deformation of the Ntraining samples. As stated in [49,60], the coefficients , ω_0_, **ω**, **ψ**_H_ and **ψ**_0_ , in the classifier h_TPSRC_(**x**) can be learned by a group of linear equations based on the training set at a very low computational cost. Detailed construction of the linear equation set with the solution of the coefficients can be found in Appendix D.

### 5.3. MARSC

The multivariate adaptive regression spline (MARS) method is a multivariable, nonlinear and nonparametric regression technique for flexible modeling of high dimensional data. The classification model simulates the complex nonlinear relationship by spline functions, which takes the form of an expansion in product spline basis functions, where the number of basic functions as well as the parameters associated with each one (product degree and knot locations) are automatically determined by the training samples [50].

MARS is not only a data-driven function regression method, but also has the advantages of accurate classification ability, which has broad applications in pattern recognition, system identification, process control. In contrast to TPSRC, it provides a computationally feasible approach that approximates the basis function subset selection procedure [50].

Given the training set $L={\left\{x_{Lt},y_{Lt}\right\}}_{t=1}^{M}$ and **x** = [*x*_1_,*x*_2_,…,*x_p_*]*^T^*, a feature vector with *p*-dimensional variable, each variable in the feature vector represents a factor, a general MARSC is as follows:

(18) $$h_{MARSC}\left(x\right)=\alpha_{0}+\sum_{i=1}^{K}\alpha_{i}\cdot H_{i}\left(x\right)=\alpha_{0}+\sum_{i=1}^{K}\alpha_{i}\cdot \overset{K_{m}}{\underset{k=1}{\Pi }}{\left[s_{km}\left(x_{v\left(k,m\right)}-t_{km}\right)\right]}_{+}$$

where *K* is the number of spline basis functions, **α** = {α_0_,α_1_,…,α*_K_*} is the output weighting value, *K_m_* is the number of factors in the *m*-th basis function *H_i_*(**x**), *s_km_* accepts only two values {+1,–1}, which indicates the sense of the truncation, *v*(*k,m*) labels the predictor variables and represents which predictor variable locating in the *k*-th section of *m*-th basis function, *t_km_* is the knot location or partition threshold. [*s_km_*(*x_v_*(*_k,m_*)–*t_km_*)]_+_ is a step polynomial, namely:

(19) $${\left[s_{km}\left(x_{v\left(k,m\right)}-t_{km}\right)\right]}_{+}=\left\{ \begin{array}{ll} s_{km}\left(x_{v\left(k,m\right)}-t_{km}\right) & s_{km}\left(x_{v\left(k,m\right)}-t_{km}\right)>0\\ 0 & others \end{array}\right.$$

MARSC function described in the Equation(18) can be expanded in a more explicit form by a simple rearrangement of terms:

(20) $$h_{MARSC}\left(x\right)=\alpha_{0}+\sum_{K_{m}=1}h_{MARSC_{i}}\left(x_{i}\right)+\sum_{K_{m}=2}^{}h_{MARSC_{ij}}\left(x_{i},x_{j}\right)+\sum_{K_{m}=3}^{}h_{MARSC_{ijk}}\left(x_{i},x_{j},x_{k}\right)+\cdots$$

where the first summation term in Equation (20) collects together all basis functions that involve only one variable (*K_m_* = 1), the second term collects together all the basic functions which involve two but only two factors, analogously, the *i*-th term collects all the basic functions that involve *i* factors.

Standard MARS uses a forward/backward stepwise strategy to produce a set of basic functions. The forward part is an iterative procedure, each of which simultaneously constructs an expanded list of basic functions to be considered and then decides which ones to enter at that step. After implementing the forward stepwise selection of basic functions, a backward procedure is applied in which the model is pruned by removing those basis functions that are associated with the smallest increase in the (least squares) goodness-of-fit. A least squares error function (inverse of goodness-of-fit) is computed. The so-called generalized cross validation (GCV) error is a measure of the goodness of fit that takes into account not only the residual error but also the model complexity as well. When the GCV reaches the least value, the best model is achieved. GCV is given by [50]:

(21) $$GCV\left(K\right)=\frac{1}{M}\frac{\sum_{i=1}^{M}{\left(y_{Li}-h_{MARSC}\left(x_{Li}\right)\right)}^{2}}{{\left(1-\frac{C\left(K\right)}{M}\right)}^{2}}$$

where *C*(*K*) = *K*(*d*/2 + 1), *d* regards as a smooth parameter, usually *d* = 3.

### 5.4. Algorithm Steps and Effectiveness Analysis

#### 5.4.1. COSC-Boosting Algorithm Steps

The main steps of the COSC-Boosting algorithm for semi-supervised classifier learning are displayed in Algorithm 1. It is noteworthy that the unlabeled samples set and the test set can overlap. In each iteration, COSC-Boosting chooses *M*’samples randomly from the unlabeled sample set, based on the labeling confidence computing, the samples who are assigned the sample labels by the complementary classifier are recorded as the candidate high confidence samples to update the classifiers in the current repeating step and the most confidential labeling samples are elected as the virtually labeled samples to augment the labeled sample set and consequently regularize the classifier learning.

**Algorithm 1.** Pseudo code description of COSC-Boosting algorithm.  COSC-Boosting**Input**:  label sample set: $L={\left\{\left(x_{t}^{L},y_{t}^{L}\right)\right\}}_{t=1}^{M},$    unlabeled data set: $U={\left\{\left(x_{t}^{U},-\right)\right\}}_{t=1}^{N}$,    maximum number of iteration: *T*    number of randomly chosen samples in the unlabeled set for classifier updating: *M*’ **Output**: the eventual classifier *f* **procedure** :   $L_{1}\leftarrow L$;  % Prepare a labeled sample set *L*_1_ for TPSRC;   $L_{2}\leftarrow L$;  % Prepare a labeled sample set *L*_1_ for TPSRC;    Create a buffer pool $U^{\prime }\leftarrow {\left\{\left(x_{i}^{U^{\prime }},-\right)\right\}}_{i=1}^{M^{\prime }}$ to save the *M*’ samples randomly chosen from *U*;    Training TPSRC *h_TPSRC_*(**x**) based on *L*_1_ .    For each $x_{i}^{U^{\prime }}\in U^{\prime }$
$x_{i}^{U\prime }\in U\prime$    $y_{x_{i}^{U\prime }}^{TPSRC}=h_{TPSRC}\left(x_{i}^{U^{\prime }}\right)$;    $y_{x_{i}^{U\prime }}^{MARSC}=h_{MARSC}\left(x_{i}^{U^{\prime }}\right)$;     If $Label\left(y_{x_{i}^{U^{\prime }}}^{TPSRC}\right)=Label\left(y_{x_{i}^{U^{\prime }}}^{MARSC}\right)$ % gain the same label from TPSRC and MARSC      $MSE\left(h_{TPSRC};x_{i}^{U^{\prime }}\right)=\sum_{x_{i}\in L_{1}}\left\{{\left[y_{i}-h_{TPSRC}\left(x_{i}\right)\right]}^{2}-{\left[y_{i}-h_{TPSRC}^{*}\left(x_{i}\right)\right]}^{2}\right\};$      $MSE\left(h_{MARSC};x_{i}^{U^{\prime }}\right)=\sum_{x_{i}\in L_{2}}\left\{{\left[y_{i}-h_{MARSC}\left(x_{i}\right)\right]}^{2}-{\left[y_{i}-h_{MARSC}^{*}\left(x_{i}\right)\right]}^{2}\right\};$     If both $MSE\left(h_{TPSRC};x_{i}^{U^{\prime }}\right)\geq 0$ and $MSE\left(h_{MARSC};x_{i}^{U^{\prime }}\right)\geq 0$       $h_{TPSRC}\leftarrow h_{TPSRC}^{*}$;   %Update $h_{TPSRC}$;       $h_{MARSC}\leftarrow h_{MARSC}^{*}$;   %Update $h_{MARSC}$;     End If      End If End For $\pi_{TPSRC}\leftarrow \phi$; If exist $MSE\left(h_{TPSRC};x_{i}^{U^{\prime }}\right)\geq 0$ % find the labeling of most confidence  ${\tilde{x}}_{TPRSC}^{U^{\prime }}\leftarrow agr\text{max}\left\{MSE\left(h_{TPSRC};x_{i}^{U^{\prime }}\right)\right\};\;y_{TPRSC}^{U^{\prime }}\leftarrow h_{TPRSC}\left({\tilde{x}}_{TPRSC}^{U^{\prime }}\right);$  $\pi_{TPSRC}\leftarrow \left\{\left({\tilde{x}}_{TPRSC}^{U^{\prime }},y_{TPRSC}^{U^{\prime }}\right)\right\}$;   End If $\pi_{MARSC}\leftarrow \phi$;  If exist $MSE\left(h_{MARSC};x_{i}^{U^{\prime }}\right)\geq 0$ % find the labeling of most confidence   ${\tilde{x}}_{MARSC}^{U^{\prime }}\leftarrow agr\text{max}\left\{MSE\left(h_{MARSC};x_{i}^{U^{\prime }}\right)\right\};\;y_{TPRSC}^{U^{\prime }}\leftarrow h_{MARSC}\left({\tilde{x}}_{MARSC}^{U^{\prime }}\right);$   $\pi_{MARSC}\leftarrow \left\{\left({\tilde{x}}_{MARSC}^{U^{\prime }},y_{TPRSC}^{U^{\prime }}\right)\right\}$;    $L_{1}\leftarrow L_{1}\cup^{\text{​}}\pi_{TPRSC};\;L_{2}\leftarrow L_{2}\cup^{\text{​}}\pi_{TPRSC};$   If neither *L*_1_ and *L*_2_ changes then directly exit the repeating;   Else   Training *h_TPRSC_*(**x**) based on *L*_1_ and *h_MARSC_*(**x**) based on *L*_2_ separately;   Reset *U*’ and Randomly select *M*’ samples from *U* with replacement to *U*’;   End If End Repeat **Output**: the ultimate classifier $f\left(x\right)=\frac{1}{2}\left(h_{TPRSC}\left(x\right)+h_{MARSC}\left(x\right)\right);$;

#### 5.4.2. Post-Processing for Product Quality Labeling

After the coefficients of the classifiers *h_TPRSC_*(**x**) and *h_MARSC_*(**x**) have been learnt, the regression values of the unlabeled points can be directly evaluated by the function described in Equations (17) and (20). For instance, for a new input image *t*_i_ with feature vector $x_{t_{i}}$, its associated quality-related output is ${\hat{y}}_{t_{i}}^{TPRSC}=h_{TPRSC}\left(x_{t_{i}}\right)$ or ${\hat{y}}_{t_{i}}^{MARSC}=h_{MARSC}\left(x_{t_{i}}\right)$. However, the output is apparently not restricted to be the labels in {+1,–1}. Hence, we should employ a threshold *T*, e.g., assigning the “medium” value zero as the classification threshold, namely, a very simple labeling rule is as follows:

(22) $$y_{t_{i}}=Label\left({\hat{y}}_{t_{i}}\right)=\{\begin{array}{c} -1\begin{array}{cc} \left(“\text{high quality}”\text{ product}\right) & \text{if }{\hat{y}}_{t_{i}}\leq T \end{array}\\ 1\begin{array}{cc} \left(“\text{other quality}”\text{ product}\right) & others \end{array} \end{array}$$

Furthermore, the eventual labeling results by the output of COSC-Boosting algorithm should be post-processed analogously as the labeling process of single classifier, namely, a thresholding processing should be carried out, in the two-class case the threshold can be set as the “medium” value zero as described in Equation (22).

#### 5.4.3. Relation to Other Algorithms

The proposed COSC-Boosting algorithm is inspired by the COREG algorithm, a co-training-style semi-supervised regression algorithm [48], whose effectiveness is demonstrated in detail. Analogously, in the learning processing of the COSC-Boosting algorithm, if and only if the newly labeled sample **x***_u_* makes the classifier more consistent with the labeled samples is set as the candidate set to regulate the classifier learning. The evaluation criterion of consistence of **x***_u_* is as follows:

(23) $$\Delta_{u}=\frac{1}{\left|\tilde{L}\right|}\sum_{x_{i}\in \tilde{L}}\left\{{\left[y_{i}-h_{l}\left(x_{i}\right)\right]}^{2}-{\left[y_{i}-h_{l}^{*}\left(x_{i}\right)\right]}^{2}\right\}$$

where *h_l_* represents TPSRC or MARSC, $h_{l}^{*}$ denotes the refined version of *h_l_* with the newly and properly labeled example ($x_{u},h_{l}\left(x_{u}\right))$. If Δ*_u_* is positive, it indicates that the refined classifier $h_{l}^{*}$ evolves towards being more consistent with the labeled sample. The labeling sample of most confidence, maximizing the value of $MSE\left(h_{l};x_{u}\right)$ is picked out to augment the labeled samples for classifier learning. The unlabeled example chosen according to the maximization of $MSE\left(h_{l};x_{u}\right)$ will result in a positive value of Δ*_u_*, as explained in [48].

Unlike the commonly-used cross validation method in semi-supervised learning for determining the label confidence of the unlabeled samples, the proposed COSC-Boosting algorithm employs two complementary classifiers for classifier learning, which does not require cross validation, nor does it require redundant views construction on the training samples. The complementarities of the classifiers with the label confidence evaluation criterion guarantee the most confident unlabeled samples in each iteration to benefit the classifier learning.

In the final formula of TPSRC in Equation (17), the Green’s function φ(*r*) relates to the labeled sample’s overall feature vector, r=∥x–$x_{i}∥,$ which is a distance metric and cannot use the single factor as well as the coordinating role or the interaction of the factors in the feature vector **x**. In other words, TPSRC establish an appropriate regression or classification model from the overall feature vector of the sample, whereas MARSC does not only consider the contribution of single factor, but also the cooperation and interaction of the factors in the feature vector. As described by Equation (20), MARSC takes full advantage of the characteristic of the sample feature information and mines the underlying complex structure information from the multidimensional feature vector, which is complementary to the TPSRC.

The COREG algorithm is more inclined to the diversities of the newly labeled samples, while the proposed COSC-Boosting algorithm doses not only consider the diversity of newly labeling samples, but also the noise suppression ability and overfitting prevention. The diversity is carried out by the selection of *M*’ unlabeled samples in each iteration for classifier updating. Since the established classifiers are complementary, the probability of misclassification on an unlabeled sample with both of the classifiers will significantly decrease, when the same tag unanimously made by the two classifiers. Hence, in terms of the problem of noisy samples, the COSC-Boosting algorithm achieves high accuracy on the labeling of unlabeled samples.

The criterion for high confident sample selection by complementary classifiers is much more stringent in the proposed COSC-Boosting algorithm, namely, only the samples gaining consistent labels by two complementary classifiers and both the two classifiers can be refined more consistently with the labeled samples are chosen as the high confident samples for classifier learning, which can effectively prevent the problem of overfitting or vibration in the learning procedure caused by the wrongly regulation based on the impertinent introduction of newly labeled samples. In the extreme situation, only a few unlabeled samples in *U* achieve consistent labels based on TPSRC and MARSC, then the Learning process COSC-Boosting algorithm degenerates to a normal ensemble of two classifiers, by consulting the results of two complementary classifiers, better classification accuracy will also be achieved.

## 6. Experimental Verification

The proposed image statistical modeling with semi-supervised learning-based product quality inspection approach is tested on a food processing factory, in which several kinds of cereal, e.g., rice, corn, are processed by dehusking, polishing, screening, grading and packaging for marketing, located in a province in south China. The proposed method is tested on the assembly line for rice processing-quality grading.

### 6.1. Overview of Visual Sensors-Based Cereal Product Quality Classification

Rice is the world’s most consumed staple food, and the predominant dietary energy source in China. Almost all of the rice processing enterprises attempt to employ OPQI technology instead of inefficient and subjective manual inspection to provide high-quality rice products. Nowadays, visual sensor-based rice quality inspection [61] has drawn wide attention.

Rice product is a typical GP. In earlier years, people tended to be more concerned about the physical properties of each individual grain, e.g., surface gloss, shape, size and other related characteristics [62], in visual sensor-based cereal quality monitoring. As addressed in the surveys [63,64], the first step is GPI segmentation, which is the foundation for GPI feature extraction [61]. Then, a kind of classifier such as artificial neural networks (ANN), support vector machines (SVM) or other supervised methods is exploited for product quality grading [65].

**Figure 4:**
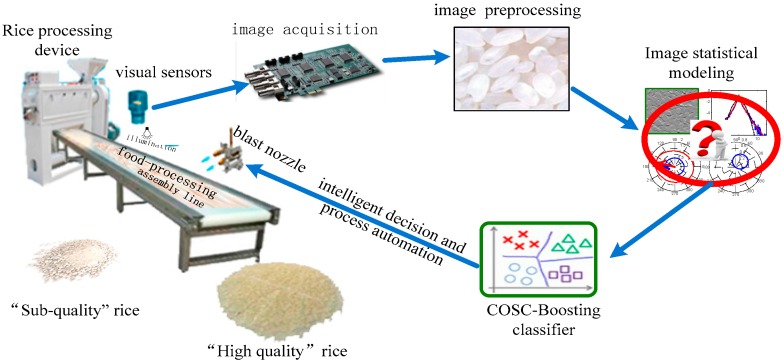
Schematic diagram of a visual inspection system forcereal food quality monitoring.

Although many experiments have verified the effectiveness of these methods and their high grading accuracies (higher than 90%), many unresolved problems restrict their practical application. For example, there is a lack of sufficiently effective and efficient GPI segmentation methods in GPI analysis. As reported in the literature, the highest record reported is only 1200 particles per minute [63]. Furthermore, in the product quality classification stage, the lack of adequate labeled samples is another barrier for classifier learning, because labeling the rice quality is fairly expensive. Figure 4 displays the schematic of a visual inspection system for rice quality monitoring.

During the rice processing, rice grains are evenly distributed on the conveyor belts. The OPQI system examines the cereal grains on the conveyor belts to identify the rice quality based on rice image features. When the cereal quality is found inferior, the actuator (blast nozzle) is automatically controlled to blast air. Then, the low-quality product is blown away from the conveyor belt for cereal food reprocessing or recovery to classify the cereal grains of different qualities and to yield high-quality rice product for consumers.

### 6.2. Rice Quality Grading

#### 6.2.1. Configuration

In the rice processingassembly line, to process the ricein parallel in the rice milling process there are multiple conveyors, among which each the conveyor belt is approximately 95mm wide. The capacity of each conveyor belt reaches 45 kg/h. The visual sensors in the OPQI system in the experiment are equipped above the conveyor belt perpendicular to the belt surface for rice quality monitoring. In the experiments, an IL-P3-2048 Linear CCD is mounted, whose pixel dimensions are 14 μm × 14 μm, and the active pixel per line is 2048 with a 20MHz data rate per tap. The F24mm/2.8 fixed-focus lens (Computar, CBC Group, Tokyo, Japan) is used, and 8-bit gray scale image frame with the pixel resolution of 2048 × 128 is captured for rice quality inspection.

According to the actual demand of the factory, we only consider a two-value classification problem. The label of product quality is defined as follows:

(24) $$y_{t}=\{\begin{array}{c} -1\\ +1 \end{array}\begin{array}{c} \text{“high quality” rice}\\ \text{“other quality” rice} \end{array}$$

A total number of 6250 samples, including rice images with corresponding rice quality labels calibrated manually, from five different rice varieties (RVs) are collected for experimental verification. The rice varieties with their corresponding number of samples in the experiment are displayed in Table 1.

In the classification experiments, given $NT_{T_{i}}$ samples of rice variety *i* are randomly selected as the labeled samples for training samples and the remainder are used as the unlabeled samples both for classifier learning and classification performance evaluation, the classification error (CE) of rice variety *i* can be calculated with the following formula:

(25) $$CE_{i}=\frac{1}{N_{i}-N_{T_{i}}}\sum_{t=1}^{N_{i}-N_{T_{i}}}\frac{\left|{\hat{y}}_{t_{i}}-y_{t_{i}}\right|}{2}*100\%$$

where ${\hat{y}}_{t_{i}}$ and $y_{t_{i}}$ are the estimated and true label of the test sample *t* of rice variety *i*. Thus, the average CE (ACE) can be evaluated with respect to the RVs (*ACE*_RV_), which does not consider the different test numbers of different RVs:

(26) $$ACE_{\text{RV}}=\left(\frac{1}{5}\sum_{i=1}^{5}CE_{i}\right)*100\%$$

And it can be also calculated with respect to the total test samples (*ACE*_TS_), which does not take into account of the RVs of the samples:

(27) $$ACE_{\text{TS}}=\frac{1}{\sum_{i=1}^{5}N_{i}-N_{T_{i}}}\sum_{i=1}^{5}\sum_{t=1}^{N_{i}-N_{T_{i}}}\frac{\left|{\hat{y}}_{t_{i}}-y_{t_{i}}\right|}{2}*100\%$$

To increase the statistical robustness of the classification results, 100 independent Monte Carlo experiments are conducted and the experimental mean value and standard deviation are recorded. The average *CE*,*ACE*_RV_ and *ACE*_TS_ with the standard deviation of the repeated experiments is recorded to evaluate the rice quality classification performance.

Regarding the image feature extraction, the optimal steerable edge and ridge GDF templates proposed by Jacob [12] is adopted to capture the omnidirectional ISS of GPI by OGDF based on the Formula (8). Six steerable GDF templates adopted in the experiment are as follows:

(28) $$T_{1}=-\sqrt{2/\pi }G_{y}$$

(29) $$T_{2}=-0.966G_{y}-0.256\sigma^{2}G_{xxy}$$

(30) $$T_{3}=-1.0655G_{y}-0.2\sigma^{2}G_{xxy}-0.042\sigma^{2}G_{yyy}$$

(31) $$T_{4}=-\sqrt{3/\left(4\pi \right)}\sigma \left(G_{yy}-G_{xx}/3\right)$$

(32) $$T_{5}=-0.204G_{yy}+0.059\sigma G_{xx}+0.063\sigma^{2}G_{yyyy}-0.194\sigma^{3}G_{xxyy}+0.024\sigma^{3}G_{xxxx}$$

(33) $$T_{6}=-\sqrt{2/\left(2+\pi +2\text{cos}\phi \right)}\left[G_{x}+\sigma \cos\left(\phi /2\right)/\sqrt{\pi }\left(G_{xx}-G_{yy}\right)\right]$$

**Figure 5:**
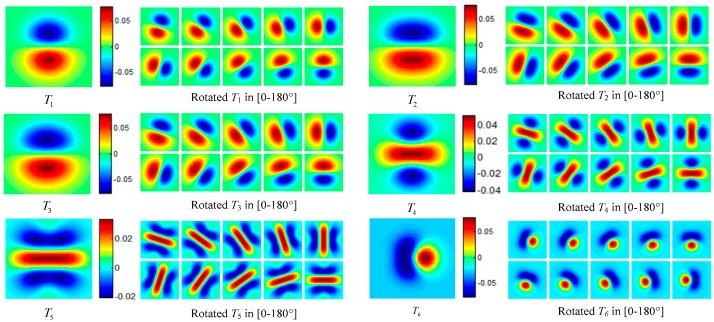
Steerable filter templates with their rotated versionsin the first two quadrants.

Among these filter templates, *T*_1_, *T*_2_ and *T*_3_ are the optimal *edge detectors* with different derivative orders of mixed GDF based on the optimization of a Canny-like criterion, *T*_4_ and *T*_5_ are the best *ridge detectors* of different derivative order, and *T*_6_ is a Wedge detector with the wedge angle φ. Templates *T*_1_~*T*_6_ with their corresponding rotated versions in [0~π] are displayed in Figure 5. The computing method of OGDF with these steerable templates is discussed in Appendix E.

A total of 60 directions is uniformly sampled in [0~π] to obtain omnidirectional ISS of GPI. Five Gaussian scales, [σ_1_,σ_2_,…,σ_5_] = [$0.5,\;\sqrt{2}$/2,1,$\sqrt{2,}2]$, are selected for the sake of extracting the multiscale ISSs under various Gaussian observation scales. Hence, we can achieve a 5 × 60 × 3 × (*N* + 1)-dimensional WD-MP feature vector from the 5 × 60 filtering responses with regard to each steerable filter template as denoted by Equation (13), where *N* denotes the number of the nonoverlapingsubimages considered in the original image. If *N* = 0, it means we do not partition the original image into subimages for local ISS feature extraction and only the global ISS information is considered.

#### 6.2.2. Classification Result and Performance Comparison

##### Experiment I: Validation Experiments

Both single steerable filter template experiments and hybrid filter template experiments are performed in this study to evaluate the rice quality classification accuracy. Table 2 displays the rice quality classification results measured in CE (%) with single different filter templates (*T*_1_~*T*_6_) of the 100 independent Monte Carlo repetition experiments.

In the experiments, the number of the sub-images for local ISS feature analysis is four, namely, one image is partitioned to four local subimages for feature extraction and the extended WD-MP features are used for rice quality classifier. In terms of the classifier learning, the label rate of the samples is 45% for classifier training and the reminding 55% samples as the unlabeled samples both used for training and classification test in each repetition experiment. In the COSC-Boosting learning, the number M′ of unlabeled samples selected with replacement for classifier learning is a constant 100.

In Table 2, *ACE*_RV,mean_ and *ACE*_TS,mean_ record the average values of *ACE*_RV_ and *ACE*_TS_ of the 100 independent repetition experiments, respectively; whereas *ACE*_RV,std_ and *ACE*_TS,std_ are the mean standard deviation values of *ACE*_RV_ and *ACE*_TS_, respectively. The column titles “mean” and “std” record the mean and standard deviation values of CE for each rice variety.

As can be seen from Table 2, when only one kind of GDF template is chosen independently for ISS feature extraction, the best classification result can be achievedwith the filter template *T*_5_, whose average classification accuracy (ACA) on the five RVs ((1 − *ACE*_RV_) × 100%) can reach 94.53% and ACA on all of the test samples ((1 − *ACE*_TS_) × 100%) is 93.08%. That the rice image is composed of a large number of locally homogeneous grains of random distribution and the ISS of rice image is determined largely on the edges and ridges of the grains in the rice image, resulting from the randomly distributed grains of locally homogeneous. In accordance with the properties of the templates, *T*_5_ is a kind of ridge detector, it can effectively extract the ridge structures as well as the edge curves of the rice grains, thus the most important ISS of the rice image can be attained. Hence, the extracted WD-MP features with GDF template *T*_5_, attaining the more essential ISS characteristics of the rice image, achieve relatively better classification results. The next best classification result can be achieved with the GDF template *T*_1_, whose ACA on the six RVs reaches 93.28%.

The worst classification accuracy comes from the GDF template *T*_3_, whose ACA on the six RVs is slightly below 90%. However, the classification accuracies on any of the five RVs basically satisfy the practical requirements. The ACAs of the six templates on the five RVs and on all the test samples are 91.95% and 90.52%, respectively.

In addition, the proposed method in the single template experiment is also quite stable, which can be apparently indicated by the low standard deviation values of CE of the independent Monte Carlo experiments on different RVs. Hence, the proposed WD-MP features on the omnidirectional ISS based on the optimal steerable edge or ridge detection templates *T*_1_ − *T*_6_ is reasonable and basically satisfying in the rice quality inspection.

The focuses of the filter templates *T*_1_ − *T*_6_ are different, e.g., *T*_1_ − *T*_3_ are dedicated to detect the edge information in images, and *T*_4_, *T*_5_ are ridge detectors, and *T*_6_ is the wedge detector, thus the combination of the filter templates can obtain more elaborate ISS characterization of GPI and consequently it can achieve better classification performance for the OPQI on the theory view. Hence, we made additional rice quality classification experiments based on the combination of multiple filter templates. The rules are the combination of an edge template from different type of detectors as well as a fully integrated template (*T*_1_ + *T*_2_ + *T*_3_ + *T*_4_ + *T*_5_ + *T*_6_) experiment. The classification results are displayed in Table 3. The table entries by the fully integrated template (FIT) are boldfaced and underlined. Apart from the results by FIT, the best results on each of the five RVs by the other combination of GDF templates are underlined and the worst results on each RV are in boldface.

Comparing the classification results of single steerable filter template experiments in Table 2 with hybrid filter template experiments in Table 3, the classification accuracies improved apparently in the hybrid filter template experiments (much lower CE). Specifically, the FIT(*T*_1_ + *T*_2_ + *T*_3_ + *T*_4_ + *T*_5_ + *T*_6_) achieve the best classification accuracy, the average CE on all of the six RVs reaches as low as 1.77% and the average CE on the all of the test samples is 3.22%, in other words, the average classification accuracy can reach as high as 98.23% on all of the six RVs, which is generally a quite high classification accuracy.

The next optimal combination is “*T*_1_ + *T*_5_”, which can also achieve a very low CE or very high classification accuracy. The relatively poor results come from the combination of *T*_2_ + *T*_4_, however, the ACA on the entire six RVs still over 93%.

In view of the standard deviation values of CE to the 100 independent repeated experiments, the statistical result from the fully integrated filter templates is about 1.6%, whereas the statistics from the combination of *T*_2_ + *T*_2_ is slightly below 1%. And even the “largest” standard deviation value of CE among the different combination experiments is less than 3%.

##### Experiment II: Parameter Selection on Classifier Performance

In this group of experiments, we are mainly concerned with the classifier performance resulting from the different parameters setting in the COSC-Boosting algorithm. In contrast to the validation experiment I, the label rate of the samples for classifier learning is changed from 10% to 60%, and the number of unlabeled samples *M*’is no long a constant, whereas the GPI feature is the proposed WD-MP feature based on the fully integrated template (FIT) as the classifier model input. Analogously, 100 independent Monte Carlo repetition experiments are conducted for robust comparison. The improvements (average improvement with the standard deviation value on the 100 independent repetition experiments) on the CE are displayed in Table 4, where the improvement is evaluated by subtracting the final CE (based on the proposed COSC-Boosting algorithm) from the initial CE, which does not perform the semi-supervised learning procedure and just performs by consulting the results from the two separated TPSC and MARSC ($\left(h_{TPRSC}\left(x\right)+h_{MARSC}\left(x\right)\right)/2)$.

The results from Table 4 can be seen clearly that the proposed COSC-Boosting algorithm performs significantly better than the simple ensemble of the two initial classifiers. In other words, that the proposed semi-supervised learning classifier can effectively exploit the underlying information of the unlabeled samples and hence it eventually greatly improves the performance of product quality grading.

##### Experiment III: Comparisons

In order to compare the performance of the proposed method with related methods, we perform rice quality classification experiments with different GPI feature extraction methods and different pattern classification methods. In the GPI feature extraction, we selected some well-known related feature extraction methods for rice quality classification test, namely, the gray level co-occurrence matrix (GLCM) [66] and the gray level run length matrix (GLRM) [66], Wavelet transform analysis (WTA) method [67], and Gabor transform (GT) method [68].

Detailed GLCM/GLRM feature extractionmethod are as follows:(a) The image intensity is quantized to 8, 32 and 64 brightness levels; (b) At each quantization scale, calculate the GLCM/GLRM matrix;(c) Based on each GLCM/GLRM matrix, we extract in a total of 14 statistics [66], e.g., energy, inertia moment, partial correlation, entropy, fineness, to constitute the spatial structural feature vector of the rice images.

WTA feature are extracted as follows: (a) Image colour space is transformed into HIS and CIE L*a*b*color spaces; (b) In each independent colour space, we conduct multi-scale image decomposition using Db4 wavelet for rice image analysis, until the coarsest scale of the image size is not less than 8 × 8; (c) In each decomposition scale, we calculate energy, colour covariance, etc. In total of 15 parameters [69] computed from the detail Wavelet decomposition coefficients.

GT feature is extracted as follows: (a) Gabor wavelets are defined by:

(34) $$\psi_{u,v}\left(Z\right)=\frac{{\left\|k_{u,v}\right\|}^{2}}{\sigma^{2}}e^{\left(-{\left\|k_{u,v}\right\|}^{2}{\left\|Z\right\|}^{2}/2\sigma^{2}\right)}\left[e^{ik_{u,v}Z}-e^{-\sigma^{2}/2}\right]$$

where *u* and *v* define the orientation and scale of Gabor kernels, $Z={\left(x,y\right)}^{T},∥▯∥$ denotes the norm operator, and the wave vector $k_{u,v}=k_{v}e^{i\phi_{u}}$, where *k_v_* = *k*_max_/*f^v^* and *φ_u_* = *πu*/8, *k*_max_ is the maximum frequency and *f* is the spacing factor between kernels in the frequency domain. (b) Five scales and eight orientations of the defined Gabor kernels are considered in the rice image analysis, namely *u* = 8, and *v* = 5, the other parameters in the Gabor kernel are defined as *σ* = 2*π*, *k*_max_ = *π*/2 and $f=\;\sqrt{2}$. (c) Statistics, mean value and standard deviation value of each magnitude response of Gabor filtering are extracted to establish a 40 × 2 dimensional feature vector as the rice image feature.

The classifier selection is another influencing factor besides the GPI feature extraction to OPQI. As addressed before, any supervised classifier can be used in this task. Two commonly-used supervised learning classifier, least squares-support vector machine (LS-SVM) and learning vector quantization-neural network(LVQ-NN) [70] classifier is used for the rice-quality grading experiment. The node number of hidden layer of LVQ-NN is determined by the best classification performance through cross-validation. The procedure of tenfold cross-validation repeated 10 times is used in the comparative experiment. By the repetitive experiments with different training samples, we find that 25 hidden layer nodes could obtain the best recognition results.

In order to achieve robust classification results, 100 independent repetition experiments are also carried out.In each repetition experiment, the training and test samples used for five different kinds of rice are identical (in this experiment, 45% of the samples are used for classifier training and the remainder is used for testing). The classification results (average improvement with the standard deviation value on the 100 independent repetition experiments) achieved by the combination of different image features with different classifiers are displayed in Table 5. The proposed WD-MP feature is extracted based on the FIT. The best classification performance (the minimum CE mean value) on each rice variety is underlined in the Table 5.

As can be seen from Table 5, regardless of the choice of different classifier, the best classification performance (minimum CE) comes from the proposed WD-MP feature on almost every rice variety. The only exception of the rice variety of RGGR, where the best classification performance is achieved by the combination of the GLCM and GLRM feature with the LS-SVM, however, it is only a little better than that of the combination of the WD-MP feature with LS-SVM. The performances based on the commonly-used GPI features, GLCM, GLRM, WTA, GT, regardless of the classifier, are actually basically comparable, in other words, no single feature extraction method performed better than the others in the GP quality classification. The average classification accuracy by the commonly-used image feature with commonly-used classifiers on every variety of rice is basically lower than 90%, whereas, the average classification accuracy will be slightly higher than 91% if we change the commonly-used image feature extraction methods to the proposed WD-MP feature. However, the classification performance based on the combination of the proposed WD-MP feature with the commonly supervised learning classifier is apparently inferior to that of the combination of WD-MP feature with COSC-Boosting classifier, which effectively exploits the unlabeled samples and the classification accuracies with the same GPI feature can reach higher than 98% on four rice varieties and the classification accuracy on the reminder rice variety is only a litter lower than 98%.

Combining the results from the validation experiment and the comparative experiment, we can draw the following conclusions:

(1) With the combination of the edge, ridge and wedge detector of GDF template, the ISS of GPI can be effectively characterized.
(2) Because the ISS of GPI is proved to conform to the WD model, the proposed WD-MP features are a generally elaborate descriptor of ISS of grain images, which are closely related to the perceptual significance of HVP.
(3) According to the presented design procedure of OGDF, the omnidirectional ISS of grain image can be effectively computed with low computation cost by the formula, which facilitates the practical applications of the proposed image statistical modeling based OPQI of product quality.
(4) The proposed COSC-Boosting algorithm is an effective semi-supervised learning algorithm based on the complementary classifiers, TPSRC and MARSC, which can effectively exploit the underlying information of the unlabeled samples and achieve much better classification performance.

In summary, OPQI based on the proposed WD-MP features integrated with the semi-supervised COSC-Boosting classifier can achieve pretty high classification accuracies with sufficient robustness, which can effectively meet the imperative demand of industrial product quality inspection.

## 7. Conclusions

A kind of image statistical modeling integrated with a semi-supervised learning method for GP quality grading is presented to facilitate the practical applications of OPQI systems. We focus on delineating the ISS features of GPIs, comprising of stochastically stacking fragments (particles) of local homogeneity, without distinctive foregrounds and backgrounds, which brings great challenges in the intelligent identification of the product qualities, e.g., rice images, fabric images.

The WD processes of ISS of these images are explained by introducing the theory of *sequential fragmentation*. The OGDFs are established with low computation complexity to attain the ISS of complex grain images. The WD-MPs of ISS are extracted as the visual features for product quality identification, which are demonstrated to be closely related to the human visual perceptual properties with great perceptual significance. In the face of the scarcity of labeled samples, a co-training-style semi-supervised classifier algorithm, named COSC-Boosting, is exploited for semi-supervised GP quality recognition, by integrating two independent classifiers, TPSRC and MARSC, with complementary nature.

The proposed GP quality grading method integrated WD-MP features with COSC-Boosting classifier is tested in the field of rice quality grading for rice processing monitoring onan industrial scale assembly line. The experimental results indicate that the proposed WD-MP features can effectively characterize the statistical distribution profiles of ISS of these intricate texture images with a large number of stochastically accumulative fragmentations. The proposed method provides an effective tool for grain image modeling and analysis and consequently lays a foundation for the intelligent perception of the product-quality on assembly lines.

## Figures and Tables

**Table 1:** Rice varieties with the corresponding sample numbers for experimental verification.

Rice Variety		Number of Samples
Chinese “see-mew” rice(CSMR)	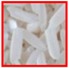	1295
Ningxia Pearl rice(NPR)	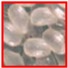	1206
Jinyou rice(JR)	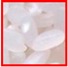	1198
Round-grain glutinous rice(RGGR)	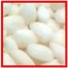	1246
Wuchang paddy aroma rice (WPAR)	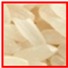	1305

**Table 2:** Rice quality classification results with a single steerable filter template.

Rvs	*T*_1_		*T*_2_		*T*_3_		*T*_4_		*T*_5_		*T*_6_	
	*mean*	*Std*	*mean*	*std*	*mean*	*std*	*mean*	*std*	*mean*	*std*	*mean*	*std*
CSMR	8.56	4.72	9.73	4.04	9.59	4.64	8.77	4.80	5.99	4.50	9.38	3.40
NPR	7.51	2.24	9.33	2.70	10.39	2.50	8.56	2.08	5.26	2.43	9.26	2.59
JR	6.84	3.37	9.13	3.18	9.93	3.60	8.35	3.74	5.52	0.89	9.12	3.1
RGGR	7.20	1.05	9.56	1.11	10.19	0.92	8.08	3.58	5.51	3.81	9.27	2.34
WPAR	6.83	1.38	9.15	1.24	10.21	1.19	8.36	1.35	5.31	1.31	9.42	1.44
Average CE	$ACE_{\text{RV},\text{mean}}=7.36$ $ACE_{\text{RV},\text{std}}=1.27$		$ACE_{\text{RV},\text{mean}}=9.38$ $ACE_{\text{RV},\text{std}}=1.06$		$ACE_{\text{RV},\text{mean}}=10.06$ $ACE_{\text{RV},\text{std}}=1.25$		$ACE_{\text{RV},\text{mean}}=8.44$ $ACE_{\text{RV},\text{std}}=1.73$		$ACE_{\text{RV},\text{mean}}=5.47$ $ACE_{\text{RV},\text{std}}=1.14$		$ACE_{\text{RV},\text{mean}}=9.27$ $ACE_{\text{RV},\text{std}}=2.24$	
	$ACE_{\text{TS},\text{mean}}=7.17$ $ACE_{\text{TS},\text{std}}=1.18$		$ACE_{\text{TS},\text{mean}}=10.98$ $ACE_{\text{TS},\text{std}}=0.97$		$ACE_{\text{TS},\text{mean}}=11.46$ $ACE_{\text{TS},\text{std}}=1.16$		$ACE_{\text{TS},\text{mean}}=9.83$ $ACE_{\text{TS},\text{std}}=1.64$		$ACE_{\text{TS},\text{mean}}=6.92$ $ACE_{\text{TS},\text{std}}=1.08$		$ACE_{\text{TS},\text{mean}}=9.42$ $ACE_{\text{TS},\text{std}}=1.88$	

**Table 3:** Rice quality classification results with combined steerable templates.

GDF Templates		CSMR	NPR	JR	RGGR	WPAR	*Average CE*
*T*_1_ + *T*_4_	*mean*	5.86	4.96	5.2	5.17	5.48	$ACE_{\text{RV},\text{mean}}=5.31$ $ACE_{\text{RV},\text{std}}=0.32$ $ACE_{\text{TS},\text{mean}}=6.73$ $ACE_{\text{TS},\text{std}}=1.50$
	*std*	6.41	2.98	2.21	3.37	3.38	
*T*_1_ + *T*_5_	*mean*	3.18	2.70	2.79	2.82	2.73	$ACE_{\text{RV},\text{mean}}=2.79$ $ACE_{\text{RV},\text{std}}=0.93$ $ACE_{\text{TS},\text{mean}}=4.28$ $ACE_{\text{TS},\text{std}}=0.91$
	*std*	2.26	2.35	2.58	1.23	2.18	
*T*_1_ + *T*_6_	*mean*	4.24	3.89	4.92	3.89	3.98	$ACE_{\text{RV},\text{mean}}=4.36$ $ACE_{\text{RV},\text{std}}=0.58$ $ACE_{\text{TS},\text{mean}}=4.88$ $ACE_{\text{TS},\text{std}}=1.21$
	*std*	2.56	2.78	3.87	2.12	3.76	
*T*_2_ + *T*_4_	*mean*	6.14	7.75	6.97	7.96	4.50	$ACE_{\text{RV},\text{mean}}=6.80$ $ACE_{\text{RV},\text{std}}=1.32$ $ACE_{\text{TS},\text{mean}}=8.04$ $ACE_{\text{TS},\text{std}}=1.50$
	*std*	6.11	2.99	2.20	4.22	3.06	
*T*_2_ + *T*_5_	*mean*	2.83	3.46	3.57	3.41	3.30	$ACE_{\text{RV},\text{mean}}=3.32$ $ACE_{\text{RV},\text{std}}=0.79$ $ACE_{\text{TS},\text{mean}}=4.84$ $ACE_{\text{TS},\text{std}}=0.77$
	*std*	2.38	2.42	2.48	1.15	2.26	
*T*_2_ + *T*_6_	*mean*	5.23	3.21	3.21	2.56	3.56	$ACE_{\text{RV},\text{mean}}=3.72$ $ACE_{\text{RV},\text{std}}=0.98$ $ACE_{\text{TS},\text{mean}}=4.24$ $ACE_{\text{TS},\text{std}}=1.24$
	*std*	2.45	2.67	3.12	1.26	2.12	
*T*_3_ + *T*_4_	*mean*	6.74	6.12	5.78	6.25	6.10	$ACE_{\text{RV},\text{mean}}=6.10$ $ACE_{\text{RV},\text{std}}=1.65$ $ACE_{\text{TS},\text{mean}}=5.26$ $ACE_{\text{TS},\text{std}}=1.54$
	*std*	6.57	2.74	2.68	3.84	3.03	
*T*_3_ + *T*_5_	*mean*	5.25	4.40	4.09	4.46	4.11	$ACE_{\text{RV},\text{mean}}=4.44$ $ACE_{\text{RV},\text{std}}=1.79$ $ACE_{\text{TS},\text{mean}}=5.40$ $ACE_{\text{TS},\text{std}}=1.61$
	*std*	7.60	2.81	2.02	3.92	3.00	
*T*_3_ + *T*_6_	*mean*	4.12	4.01	3.89	4.23	3.76	$ACE_{\text{RV},\text{mean}}=3.91$ $ACE_{\text{RV},\text{std}}=0.28$ $ACE_{\text{TS},\text{mean}}=5.24$ $ACE_{\text{TS},\text{std}}=2.61$
	*std*	2.40	3.45	2.68	3.12	2.12	
*T*_4_ + *T*_6_	*mean*	4.56	5.34	4.89	5.23	4.36	$ACE_{\text{RV},\text{mean}}=4.77$ $ACE_{\text{RV},\text{std}}=0.45$ $ACE_{\text{TS},\text{mean}}=4.64$ $ACE_{\text{TS},\text{std}}=1.89$
	*std*	5.34	2.56	5.34	3.12	2.89	
*T*_5_ + *T*_6_	*mean*	3.42	3.12	4.23	3.98	2.68	$ACE_{\text{RV},\text{mean}}=3.38$ $ACE_{\text{RV},\text{std}}=0.61$ $ACE_{\text{TS},\text{mean}}=6.24$ $ACE_{\text{TS},\text{std}}=2.61$
	*std*	3.45	2.45	3.45	2.56	2.45	
*T*_1_+ *T*_2_*+ T*_3_*+ T*_4_ + *T*_5_ + *T*_6_	*mean*	1.67	1.52	1.56	1.73	1.86	$ACE_{\text{RV},\text{mean}}=1.77$ $ACE_{\text{RV},\text{std}}=1.62$ $ACE_{\text{TS},\text{mean}}=3.22$ $ACE_{\text{TS},\text{std}}=1.53$
	*std*	5.56	3.31	2.15	3.64	3.67	

**Table 4:** Improvement (%) of rice quality classification with different label rate (LRs).

Parameter Setting		Rice Variety				
		CSMR	NPR	JR	RGGR	WPAR
LR = 10%	*M*’ = 50	14.22 ± 4.32	14.32 ± 2.45	12.17 ± 3.21	14.4 ± 2.22	16.43 ± 5.30
	*M*’ = 80	12.18 ± 2.34	16.23 ± 4.82	13.14 ± 3.08	16.25 ± 3.67	15.34 ± 3.45
	*M*’ = 120	18.64 ± 3.02	16.88 ± 2.21	15.23 ± 2.58	17.12 ± 3.45	16.67 ± 2.34
LR = 20%	*M*’ = 50	13.22 ± 2.84	10.14 ± 4.52	8.72 ± 4.56	9.32 ± 5.69	10.23 ± 2.48
	*M*’ = 80	14.56 ± 3.45	12.21 ± 3.42	12.34 ± 4.32	8.67 ± 2.34	12.23 ± 5.09
	*M*’ = 120	14.67 ± 2.13	12.24 ± 1.22	12.62 ± 3.21	12.12 ± 3.46	13.23 ± 1.98
LR = 30%	*M*’ = 50	10.34 ± 3.45	7.68 ± 3.42	6.45 ± 1.23	5.68 ± 3.45	8.98 ± 3.46
	*M*’ = 80	12.23 ± 2.45	8.68 ± 2.12	4.56 ± 0.98	6.12 ± 2.34	9.08 ± 2.34
	*M*’ = 120	14.56 ± 3.08	9.68 ± 1.23	5.89 ± 1.24	6.02 ± 1.23	10.02 ± 1.02
LR = 40%	*M*’ = 50	8.56 ± 3.20	8.45 ± 3.45	2.34 ± 3.46	2.34 ± 1.23	5.23 ± 2.34
	*M*’ = 80	7.45 ± 2.34	9.02 ± 2.46	4.56 ± 2.35	3.45 ± 1.46	4.56 ± 2.00
	*M*’ = 120	8.45 ± 1.86	9.06 ± 1.34	3.45 ± 1.27	2.89 ± 1.04	4.89 ± 1.06
LR = 50%	*M*’ = 50	2.34 ± 2.34	6.12 ± 2.45	5.68 ± 3.45	4.56 ± 2.13	4.56 ± 3.45
	*M*’ = 80	3.45 ± 2.14	7.24 ± 1.56	6.12 ± 2.13	4.69 ± 3.08	5.68 ± 2.34
	*M*’ = 120	4.04 ± 1.28	7.02 ± 2.21	5.89 ± 2.02	6.78 ± 1.13	6.87 ± 2.01
LR = 60%	*M*’ = 50	4.25 ± 2.32	4.89 ± 3.45	3.45 ± 2.34	0.31 ± 2.34	5.32 ± 3.56
	*M*’ = 80	4.02 ± 1.28	3.24 ± 2.45	2.45 ± 0.98	1.23 ± 2.01	6.23 ± 2.34
	*M*’ = 120	5.02 ± 1.86	4.52 ± 2.34	5.46 ± 1.56	2.34 ± 1.24	5.89 ± 0.96

**Table 5:** CE(%) of rice quality grading with different GPI features and different classifiers

Method	Rice variety				
	CSMR	NPR	JR	RGGR	WPAR
GLCM + LS-SVM	12.88 ± 2.68	14.34 ± 4.23	11.45 ± 3.45	12.23 ± 2.34	11.34 ± 3.45
GLRM + LS-SVM	13.46 ± 3.23	12.23 ± 3.45	10.34 ± 1.23	13.34 ± 4.23	12.89 ± 3.43
WTA + LS-SVM	16.34 ± 3.32	11.89 ± 2.45	12.23 ± 2.67	12.45 ± 4.42	13.23 ± 3.46
GT + LS-SVM	11.34 ± 3.78	12.23 ± 4.12	14.23 ± 3.45	10.89 ± 2.56	12.90 ± 3.67
GLCM + LVQ-NN	14.34 ± 3.12	12.34 ± 4.56	11.09 ± 2.45	10.23 ± 2.56	11.87 ± 2.78
GLRM + LVQ-NN	13.62 ± 1.34	11.89 ± 2.34	10.02 ± 2.34	11.02 ± 2.12	12.23 ± 3.46
WTA + LVQ-NN	15.24 ± 3.56	12.89 ± 2.34	9.89 ± 3.45	12.34 ± 2.34	11.34 ± 2.48
GT + LVQ-NN	10.89 ± 3.98	13.12 ± 2.56	10.12 ± 4.34	11.23 ± 3.82	10.23 ± 3.45
(GLCM + GLRM)+LS-SVM	10.76 ± 4.23	11.12 ± 3.45	9.89 ± 2.87	8.02 ± 4.45	8.98 ± 3.69
(GLCM + GLRM)+LVQ-NN	11.23 ± 3.24	10.67 ± 4.24	8.78 ± 4.32	9.89 ± 2.45	9.92 ± 3.12
(*T*_1_ + *T*_2_*+ T*_3_*+ T*_4_ + *T*_5_ + *T*_6_)+ LS-SVM	8.92 ± 3.24	7.45 ± 2.34	8.82 ± 3.45	8.12 ± 2.34	7.89 ± 3.12
(*T*_1_ + *T*_2_*+ T*_3_*+ T*_4_ + *T*_5_+ *T*_6_) + LVQ-NN	9.03 ± 3.45	8.45 ± 4.34	7.82 ± 2.48	8.46 ± 3.40	7.66 ± 3.45

## Appendix A. WD Process of ISS

As stated in [51], any object in the viewing field fragments the scene into two regions, the internal (foreground or object) and external region (background).The visual appearance of the field of view stays stable when sufficient objects separaterandomlythe scene into a great number of fragmentations or particles [51]. The mixture of the shapes, edges, cast shadows with their distributions or organizations of the fragmentations result in the visual appearance of the ISS.

If the resolving power of the visual sensors is high enough, abundantfragments of local homogeneity with plenty details of the ISSare captured. We refer to small image patches, fragments (particles) or textons like edge parts, blobs and corners, spots as the visual details, each of which is perceived as an independent visual pattern with a consistent illumination contrast.

If the resolving power of the visual sensors decreases, adjacent fragmentations combine and merge as coarse fragmentations. On the contrary, if the resolvingpower of the visual sensors is enhanced, the coarse structure breaks up into fine structures. Therefore, the distribution of the local fragment particles in the visual image is actually equivalent to a single-event fragmentation process of continued comminution in the ore grinding process [51], which is well studied and can be characterized by the *sequential fragmentation* theory.

According to the theory of *sequential fragmentation*, the probability distribution of the illumination contrastof local fragmentation in grain images showsa power-law distribution at the assumption that small details are occurring more often in an image than large structures [53,54]. It can be described by the following formula:

(A1) $$f\left(x^{\prime }\to x\right)={\left(\frac{x-\mu }{\beta }\right)}^{\lambda -1}$$

where $x^{\prime }\to x$ represents the process of decomposing a coarse fragment structure *x*’ into fine fragment structures *x*; *μ* means the average mass of the particles in the mill of the ore grinding processes, which in this study can be represented by the average illuminant contrast of the fragments(particles) in the image; *β* is a scale parameter, related to the “width” of the contrast of the local fragments; *λ* is a shape parameter, controlling the distribution shape and satisfying *λ* ≥ 0, which is related to the particle size. In accordance with the real phenomenon that small details are occurring more often, the parameter *λ* increases, as the particle size becomes smaller.

Since the visual image is composed of diverse local fragments, the statistical distribution of the contrast of the fragment structures is the result of the integral over the various power-laws patches caused by each coarse particle [35,51]:

(A2) $$n\left(x\right)=c\int_{x}^{\infty }n\left(x^{\prime }\right)f\left(x^{\prime }\to x\right)dx^{\prime }$$

where *n*(*x*) indicates the number offragmentations of homogeneous regions withilluminant contrast between *x* and *x* + *dx*; *n*(*x*) performs statistics of all the particles with the contrast of *x*’ > *x* in the grain image. Substituting Equation (A1) into Equation (A2) and letting *c* = 1/*β*, *n*(*x*) can be computed by solving the following equation:

(A3) $$n\left(x\right)={\left(\frac{x-\mu }{\beta }\right)}^{\lambda -1}\int_{x}^{\infty }n\left(x^{\prime }\right)d\left(\frac{x^{\prime }}{\beta }\right)$$

By solving Equation (A3), it can be seen thatthe integration over a sufficient number of power-laws yields a typical WD [51,54]:

(A4) $$n\left(x\right)=N_{T}{\left(\frac{x-\mu }{\beta }\right)}^{\lambda -1}\cdot e^{-\frac{1}{\lambda }{\left(\frac{x-\mu }{\beta }\right)}^{\lambda }}$$

where $N_{T}=\int_{0}^{\infty }n\left(m\right)dm$ is a normalized parameter.

The resolving power of the visual sensor in real applications cannot be infinite, the fragmentation process of the local particles will inevitably cease. After the particle details tend to be stable, and the special visual appearance of ISS exhibits. Hence, the statistical distributions of ISS just correspond to the fragmentswith local contrast larger than *x*. Therefore, the statistical distribution model of ISSI can be described by the WD model of integral form. Its probability density model is given by:

(A5) $$f\left(x;\mu ,\lambda ,\beta \right)=N\left(>x\right)=\int_{x}^{\infty }n\left(x\prime \right)dx^{\prime }=Ce^{-\frac{1}{\lambda }{\left|\frac{x-\mu }{\beta }\right|}^{\lambda }}$$

where $C=1/\int_{-\infty }^{+\infty }e^{-\frac{1}{\lambda }│\frac{x-\mu }{\beta }{│}^{\lambda }}dx=\lambda /\left[2\lambda^{\frac{1}{\lambda }}\beta \Gamma \left(\frac{1}{\lambda }\right)\right]$ is a normalized constant, only related to the model parameter *λ* and *β* and Γ(*x*) is the gamma function.

## Appendix B. Parameter Estimation of WD Model

Given **X** = {*x*_1_,*x*_2_,…,*x_n_*} is the sampling data from an integral-form WD variable. WD Model parameters *μ*, *λ* and *β* can be estimated by the maximum likelihood estimation (MLE) method. The corresponding log-likelihood function $\text{ln}L\left(X;\hat{\mu },\hat{\lambda },\hat{\beta }\right)$ indicates how well the model describes the sampling data:

(B1) $$\ln L\left(X;\hat{\mu },\hat{\lambda },\hat{\beta }\right)=\ln\prod_{i=1}^{n}\frac{\hat{\lambda }}{2{\hat{\lambda }}^{\frac{1}{\hat{\lambda }}}\hat{\beta }\Gamma \left(\frac{1}{\hat{\lambda }}\right)}e^{-\frac{1}{\hat{\lambda }}{\left|\frac{x_{i}-\hat{\mu }}{\hat{\beta }}\right|}^{\hat{\lambda }}}$$

where $\hat{\mu },\hat{\lambda },\hat{\beta }$ represent the estimation values of WD Model parameters *μ*, *λ* and *β* . The expansion of $\text{ln}L\left(X;\hat{\mu },\hat{\lambda },\hat{\beta }\right)$ is:

(B2) $$\ln L\left(X;\hat{\mu },\hat{\lambda },\hat{\beta }\right)=\sum_{i=1}^{N}\left[\ln\hat{\lambda }-\ln2-\frac{1}{\hat{\lambda }}\ln\hat{\lambda }-\ln\hat{\beta }-\ln\Gamma \left(\frac{1}{\hat{\lambda }}\right)\right]-\sum_{i=1}^{N}\left[\frac{1}{\hat{\lambda }}{\left|\frac{x_{i}-\hat{\mu }}{\hat{\beta }}\right|}^{\hat{\lambda }}\right]$$

The model parameters can be estimated by setting the partial derivative of $\text{ln}L\left(X;\hat{\mu },\hat{\lambda },\hat{\beta }\right)$ with respect to $\hat{\mu },\hat{\;\lambda }$ and $\hat{\beta }$ be equal to zero, respectively. Thus we can obtain:

(B3) $$\frac{\partial }{\partial \hat{\beta }}\ln L\left(X;\hat{\mu },\hat{\lambda },\hat{\beta }\right)=-\frac{n}{\hat{\beta }}+\frac{1}{\hat{\beta }}\sum_{i=1}^{n}{\left|\frac{x_{i}-\hat{\mu }}{\hat{\beta }}\right|}^{\hat{\lambda }}=0$$

(B4) $$\frac{\partial }{\partial \hat{\lambda }}\ln L\left(X;\hat{\mu },\hat{\lambda },\hat{\beta }\right)=\frac{1}{{\hat{\lambda }}^{2}}\left[\hat{\lambda }n+n\ln\hat{\lambda }-n+n\Phi \left(\frac{1}{\hat{\lambda }}\right)+\sum_{i=1}^{n}{\left|\frac{x_{i}-\hat{\mu }}{\hat{\beta }}\right|}^{\hat{\lambda }}\right]-\frac{1}{\hat{\lambda }}\sum_{i=1}^{n}\left[{\left|\frac{x_{i}-\hat{\mu }}{\hat{\beta }}\right|}^{\hat{\lambda }}\ln\left|\frac{x_{i}-\hat{\mu }}{\hat{\beta }}\right|\right]=0$$

(B5) $$\frac{\partial }{\partial \hat{\mu }}\ln L\left(X;\hat{\mu },\hat{\lambda },\hat{\beta }\right)=\sum_{i=1;x_{i}\geq \hat{\mu }}^{n}\frac{{\left|x_{i}-\hat{\mu }\right|}^{\hat{\lambda }}}{{\hat{\beta }}^{\hat{\lambda }}}-\sum_{i=1;x_{i}<\hat{\mu }}^{n}\frac{{\left|\hat{\mu }-x_{i}\right|}^{\hat{\lambda }}}{{\hat{\beta }}^{\hat{\lambda }}}=0$$

where Φ(●) is the digamma function, $\Phi \left(x\right)=\frac{d}{dx}\text{ln}\Gamma \left(x\right)=\frac{d}{dx}\Gamma \left(x\right)/\Gamma \left(x\right)$.

Since we cannot obtain the closed-form solution from the Equation set (B3)–(B5), we use an iterative procedure for numerically searching the maximum value of the log-likelihood function $\text{ln}L\left(X;\hat{\mu },\hat{\lambda },\hat{\beta }\right)$ to get the numerical solution of the model parameters $\hat{\mu },\;\hat{\lambda }$ and $\hat{\beta }$. The kernel principle of the iterative procedure is derived from the Nelder-Mead simplex algorithm [55], which only uses the function values without any derivative information. This maximum value-searching method is identical to the minimum value search problem by multiplying –1 by the log-likelihood function $\text{ln}L\left(X;\hat{\mu },\hat{\lambda },\hat{\beta }\right).$ For convenience, the negative log-likelihood function is denoted as $T\left(X;\hat{\theta }\right)=-lnL\left(X;\hat{\mu },\hat{\lambda },\hat{\beta }\right)$, where $\hat{\theta }$ represents the parameter vector. The initialization parameter vector is denoted as ${\hat{\theta }}_{0},\;{\hat{\theta }}_{0}=\left[{\hat{\mu }}_{0},{\hat{\lambda }}_{0},{\hat{\beta }}_{0}\right]$. The procedure for the statistical model parameter estimation is as follows:

(1) *Initiation*:
  (a) *A simplex of four points is generated for the 3D parameter vector* θ = [*θ*_0_;*θ*_1_;*θ*_2_;*θ*_3_]*, where θ_i_*(*i* > 0) *is assigned as the initial value of θ*_0_ *by adding a% of each component θ*_0_(*i*),
  (b) Four scalar parameters ρ, χ, γ and δ and the terminal threshold tol X are initiated.
  (c) *The subscripts of the simplex points are reordered from the lowest function value to highest function value with i, i = 1, 2, 3, 4, with the new subscript of* θ, $T\left(X;\theta_{1}\right)\leq T\left(X;\theta_{2}\right)\leq T\left(X;\theta_{3}\right)\leq T\left(X;\theta_{4}\right).$
(2) *Iterative process*:

*Four-point convergence is repeated, e.g., $\max\left\{∥\theta_{i}-\theta_{j}{∥}_{i,j=1,2,3,4}\leq tolX\right\},$ then obtain the estimation $\hat{\theta }=\theta_{i}$. The repeated procedure is as follows:*

(a) $\theta_{r}=\left(1+\gamma \right)\bar{\theta }-\gamma \theta_{4},\;$ *where* $\bar{\theta }=mean\left(\theta \right)$
  *If* $T\left(X;\theta_{r}\right)<T\left(X;\theta_{1}\right);\;$
(b) $\;\theta_{e}=\left(1+\rho \chi \right)\bar{\theta }-\rho \chi \theta_{n+1}$
  *If* $\;T\left(X;\theta_{e}\right)<T\left(X;\theta_{r}\right);\;\theta_{4}=\theta_{e\;}\%\;accept\;\theta_{e}$
  $else\;\theta_{4}=\theta_{r}\;;\%\;accept\;\theta_{r}$
  end
(c) *elseif* $T\left(X|\theta_{r}\right)<T\left(X|\theta_{3}\right)\;\theta_{3}=\theta_{r};\;\%\;accept\;\theta_{r}$
  *elseif* $\;T\left(X|\theta_{r}\right)<T\left(X|\theta_{4}\right)\;\theta_{c}=\left(1+\gamma \rho \right)\bar{\theta }-\gamma \rho \theta_{n+1}$
   *If* $T\left(X;\theta_{c}\right)<T\left(X;\theta_{r}\right);\;\theta_{4}=\theta_{c\;}$
   *else goto **shrink**;*
   *end*
  else
     $\theta_{cc}=\left(1-\gamma \right)\bar{\theta }-\gamma \theta_{4}$
  *If* $T\left(X;\theta_{cc}\right)<T\left(X;\theta_{4}\right);\;\theta_{4}=\theta_{cc\;}$
  else goto**shrink**; end
  end
  end
  ***shrink:*** *for j = 2:4* $\theta_{j}=\theta_{1}+\left(\theta_{j}-\theta_{1}\right)$ *end*
  end
(d) *The subscripts of the simplex points are reordered from lowest function value to the highest function value* $\left[T\left(X\text{|θ}\right),j\right]=sort\left(T\left(X\text{|θ}\right)\right),\theta_{new}=\theta \left(j\right)$

The convergence properties of this procedure are presented in the study [55]. In the original report, the four scalar parameters can be set asρ = 1, χ = 2, γ = 0.5, and δ = 0.5. In practical applications, this procedure converges rapidly near the minimum position of the negative log-likelihood function. Hence, for fast searching, the initial model parameter θ_0_ is set as the empirical value close to the extremum, namely, ${\hat{\mu }}_{0}=median\left(X\right)$, ${\hat{\lambda }}_{0}=2\left[2\frac{\frac{1}{n}\sum_{i=1}^{n}{\left(x_{i}-\bar{x}\right)}^{4}}{{\left(\frac{1}{n}\sum_{i=1}^{n}{\left(x_{i}-\bar{x}\right)}^{2}\right)}^{3}}-3\right],\left[\frac{\frac{1}{n}\sum_{i=1}^{n}{\left(x_{i}-\bar{x}\right)}^{4}}{{\left(\frac{1}{n}\sum_{i=1}^{n}{\left(x_{i}-\bar{x}\right)}^{2}\right)}^{3}}-3\right]+1,\;{\hat{\beta }}_{0}={\left(\sum_{i=1}^{n}│x_{i}-\mu_{0}{│}^{\lambda_{0}}/n\right)}^{\lambda_{0}}.$

## Appendix C. Derivation process of the weighted coefficient $\alpha_{m,j}$

As stated in [58,59], the design and steer of the steerable filter can be better explained in the Fourier domain. Hence, we transform the rotated filter $G_{\kappa ,\sigma }\left(XR_{\theta }\right)$ into the Fourier domain to facilitate computing, and we can then obtain [59]:

(C1) $$\begin{array}{l} ℑ\left(G_{K,\sigma }\left(XR_{\theta }\right)\right)=\sum_{m=1}^{k}\sum_{i=0}^{m}k_{m,i}{\left(j\omega_{x}\cos\theta +j\omega_{y}\sin\theta \right)}^{i}{\left(-j\omega_{x}\sin\theta +j\omega_{y}\cos\theta \right)}^{m-i}G_{\sigma }\left(\omega_{x,}\omega_{y}\right)\\ =\sum_{m=1}^{k}\sum_{i=0}^{m}k_{m,i}\left\{\sum_{t=0}^{i}C_{i}^{t}{\left(j\omega_{x}\cos\theta \right)}^{t}{\left(j\omega_{y}\sin\theta \right)}^{i-t}\right\}\left\{\sum_{l=0}^{m-i}C_{m-i}^{l}{\left(-j\omega_{x}\sin\theta \right)}^{l}{\left(j\omega_{y}\cos\theta \right)}^{m-i-l}\right\}{\hat{G}}_{\sigma }\left(\omega_{x},\omega_{y}\right)\\ =\sum_{m=1}^{k}\sum_{i=0}^{m}k_{m,i}\sum_{t=0}^{i}\sum_{l=0}^{m-i}{\left(-1\right)}^{l}C_{i}^{t}C_{m-i}^{l}{\left(\cos\theta \right)}^{t+m-i-l}{\left(\sin\theta \right)}^{i-t+l}\underset{ℑ\left(\frac{\partial^{t+l}}{\partial x}\frac{\partial^{m-\left(t+l\right)}}{\partial y}G_{\sigma }\left(x,y\right)\right)}{\underset{︸}{{\left(j\omega_{x}\right)}^{t+l}{\left(j\omega_{y}\right)}^{m-\left(t+l\right)}{\hat{G}}_{\sigma }\left(\omega_{x},\omega_{y}\right)}} \end{array}$$

where ℑ(f(x)) means the Fourier transform of the function $f\left(x\right);C_{m}^{n}$ is the binomial coefficient, $C_{m}^{n}=\frac{m!}{n!\left(m-n\right)!};\;{\hat{G}}_{\sigma }\left(\omega_{x},\omega_{y}\right)$ is the Fourier transform of the Gaussian function $G_{\sigma }\left(x,y\right).$ According to the convolution theorem, we can perform Fourier transform on both sides of Equation (8), and then we can obtain:

(C2) $$\begin{array}{l} ℑ\left\{I\left(X\right)*G_{\kappa ,\sigma }\left(XR_{\theta }\right)\right\}=\hat{I}\left(\omega_{x},\omega_{y}\right)ℑ\left\{G_{\kappa ,\sigma }\left(XR_{\theta }\right)\right\}\\ =\hat{I}\left(\omega_{x},\omega_{y}\right)\sum_{m=1}^{k}\sum_{i=0}^{m}k_{m,i}\sum_{t=0}^{i}\sum_{i=0}^{m-i}{\left(-1\right)}^{l}C_{i}^{t}C_{m-i}^{l}{\left(\cos\theta \right)}^{t+m-i-l}{\left(\sin\theta \right)}^{i-t+l}ℑ\left(\frac{\partial^{t+l}}{\partial x}\frac{\partial^{m-\left(t+l\right)}}{\partial y}G_{\sigma }\left(x,y\right)\right) \end{array}$$

We then perform inverse Fourier transformation on the formula (46) and we achieve the time domain expression of $\left(X\right)*G_{\kappa ,\sigma }\left(XR_{\theta }\right)$:

(C3) $$\begin{array}{l} I\left(X\right)*G_{\kappa ,\sigma }\left(XR_{\theta }\right)={ℑ}^{-1}\left\{ℑ\left\{I\left(X\right)*G_{\kappa ,\sigma }\left(XR_{\theta }\right)\right\}\right\}\\ =\sum_{m=1}^{k}\sum_{i=0}^{m}k_{m,i}\sum_{t=0}^{i}\sum_{i=0}^{m-i}{\left(-1\right)}^{l}C_{i}^{t}C_{m-i}^{l}{\left(\cos\theta \right)}^{t+m-i-l}{\left(\sin\theta \right)}^{i-t+l}{ℑ}^{-1}\left\{ℑ\left\{I\left(x,y\right)\right\}ℑ\left(\frac{\partial^{t+l}}{\partial x}\frac{\partial^{m-\left(t+l\right)}}{\partial y}G_{\sigma }\left(x,y\right)\right)\right\}\\ =\sum_{m=1}^{k}\sum_{i=0}^{m}k_{m,i}\sum_{t=0}^{i}\sum_{i=0}^{m-i}{\left(-1\right)}^{l}C_{i}^{t}C_{m-i}^{l}{\left(\cos\theta \right)}^{t+m-i-l}{\left(\sin\theta \right)}^{i-t+l}I_{m,m-\left(t+l\right)}\left(X\right) \end{array}$$

Make a contrast of the formula (C3) and (8), if we set *j* = *m* –(*t + l*) where 0 ≤ *j* ≤ *m*, then:

(C4) $$I\left(X\right)*G_{\kappa ,\sigma }\left(XR_{\theta }\right)=\sum_{m=1}^{k}\sum_{j=0}^{m}I_{m,j}\left(X\right)\underset{\alpha_{m,j}}{\underset{︸}{\sum_{i=0}^{m}k_{m,i}\sum_{t=0}^{i}\sum_{l=0}^{m-i}{\left(-1\right)}^{l}C_{i}^{t}C_{m-i}^{l}{\left(\cos\theta \right)}^{t+m-i-l}{\left(\sin\theta \right)}^{i-t+l}}}$$

Consequently, we can obtain $\alpha_{m,j}$ as the following expression [59]:

(C5) $$\alpha_{m,j}=\sum_{i=0}^{m}k_{m,i}\sum_{t=0}^{i}\sum_{l=0}^{m-i}{\left(-1\right)}^{l}C_{i}^{t}C_{m-i}^{l}{\left(\cos\theta \right)}^{t+m-i-l}{\left(\sin\theta \right)}^{i-t+l}$$

## Appendix D. Solution of TPSRC Coefficient

By substituting the *N* training samples ${\left(x_{t},y_{t}\right\}}_{t=1}^{N}$ into the Equation (17), we obtain *N* equations, which can be expressed with matrix form:

(D1) $$\left[\begin{array}{cccc} 1_{H} & x_{H}^{T} & K_{HH} & K_{HO}\\ 1_{O} & x_{O}^{T} & K_{OH} & K_{OO} \end{array}\right]\left[\begin{array}{c} \omega_{0}\\ \omega \\ \psi_{H}\\ \psi_{O} \end{array}\right]=\left[\begin{array}{c} e_{H}\\ -e_{O} \end{array}\right]$$

where $\omega_{0}\epsilon R^{1},\text{ and }\omega ={\left[\omega_{1},\omega_{2,\;}\ldots ,\omega_{k}\right]}^{T}\epsilon R^{k},\;\Psi_{H}={\left[\psi_{1}^{H},\psi_{2}^{H},\ldots ,\psi_{M}^{H}\right]}^{T}\in R^{M},\;\Psi_{O}={\left[\psi_{1}^{O},\psi_{2}^{O},\ldots ,\psi_{M}^{O}\right]}^{T}\in R^{N-M}.$ These are the parameters to be learned in Equation (D1). In the coefficient matrix, **K***_HH_*, **K***_HO_*, **K***_OH_* and **K***_OO_* attain the values of Green’s function, where **K***_HH_* and **K***_OO_* are symmetrical matrixes, and $K_{HO}=K_{OH}^{T};\;x_{H}=\left[x_{1}^{H},x_{2}^{H},\ldots ,x_{M}^{H}\right]\in R^{k\times M},$ capturing the samples points in $\Omega^{H},$ andsimilarly, $x_{O}=\left[x_{1}^{O},x_{2}^{O},\ldots ,x_{M}^{O}\right]\in R^{k\times \left(N-M\right)},$ collecting the information from ${℧}^{O}$. In Equation (D1), $e_{H}={\left[1,1,\ldots ,1\right]}^{T}\in R^{M}$, and $e_{O}={\left[1,1,\ldots ,1\right]}^{T}\in R^{N-M}.$

It is worth noting that there are only *N* equations in Equation (D1), whereas 1 + *k* + *N* parameters should be solved in the spline function *f* for pattern classification. According to the conditions of positive definite functions [60], which relate to the uniqueness of the splines, other *d* equations can be introduced, namely:

(D2) $$\sum_{j=1}^{M}\psi j^{H}\phi j^{H}\left(x\right)+\sum_{j=1}^{N-M}\psi j^{O}\phi i^{O}\left(x\right)=0,i\in \left[1,2,\cdots ,d\right]$$

In the case of linear polynomial space, Equation (D2) can be rewritten with *k* + 1 equations in matrix form as follows:

(D3) $$\left[\begin{array}{cc} e_{H}^{T} & e_{O}^{T}\\ x_{H} & x_{O} \end{array}\right]\left[\begin{array}{c} \psi_{H}\\ \psi_{O} \end{array}\right]=0$$

Thus, we can achieve an expanded equation set as denoted in the combinationof Equations (D3) and (D1):

(D4) $$\left[\begin{array}{cccc} 1_{H} & x_{H}^{T} & K_{HH}+\xi I_{H} & K_{HO}\\ 1_{O} & x_{O}^{T} & K_{OH} & K_{OO}+\xi I_{O}\\ 0 & 0 & e_{H}^{T} & e_{O}^{T}\\ 0 & 0 & x_{H} & x_{O} \end{array}\right]\left[\begin{array}{c} \omega_{0}\\ \omega \\ \psi_{H}\\ \psi_{O} \end{array}\right]=\left[\begin{array}{c} e_{H}\\ -e_{O} \end{array}\right]$$

where the addition items *ξ***I***_H_* and *ξ***I***_O_* in the coefficient matrix are used to control the smoothness of the spline near scattered training samples, and $I_{H}\in R^{M*M}$ and $I_{O}\in R^{\left(N-M\right)\times \left(N-M\right)}$ are two identity matrices; *ξ* is usually set a positive number value to avoid the possible over-fitting of the spline function.

By the linear equation described in (D4), we can easily compute the coefficients of the TPSRC. As stated in [49], in the practical computation, **K***_HH_* and **K***_OO_* should be modified by a regularization parameter *λ*, namely, which are replace by **K***_HH_* + λ**I***_H_* and **K***_OO_* + λ**I***_O_*, where the regularization parameter *λ* governs the amount of smoothness of the spline near the scattered sample points. *λ* is usually assigned a positive value to avoid over-fitting.

## Appendix E. Rotated Filter Templates

(E1) $$T_{1}\left(XR_{\theta }\right)=-\sqrt{\frac{2}{\pi }}\sin\left(\theta \right)G_{x}+\sqrt{\frac{2}{\pi }}\cos\left(\theta \right)G_{y}$$

(E2) $$\begin{array}{l} T_{2}\left(XR_{\theta }\right)=0.966\sin\left(\theta \right)G_{x}-0.966\cos\left(\theta \right)G_{y}+0.256\sigma^{2}\cos^{2}\left(\theta \right)\sin\left(\theta \right)G_{xxx}+\\ \left[-0.256\sigma^{2}\cos^{3}\left(\theta \right)+0.512\sigma^{2}\cos\left(\theta \right)\sin^{2}\left(\theta \right)+0.256\sigma^{2}\sin^{3}\left(\theta \right)\right]G_{xxy}-\\ -0.512\sigma^{2}\cos^{2}\left(\theta \right)\sin\left(\theta \right)G_{xyy}-0.256\sigma^{2}\cos\left(\theta \right)\sin^{2}\left(\theta \right)G_{yyy} \end{array}$$

(E3) $$\begin{array}{l} T_{3}\left(XR_{\theta }\right)=1.0655\sin\left(\theta \right)G_{x}-1.0655\cos\left(\theta \right)G_{y}+\\ \left[0.042\sigma^{2}\sin^{3}\left(\theta \right)+0.2\sigma^{2}\cos^{2}\left(\theta \right)\sin\left(\theta \right)\right]G_{xxx}-\\ \left[0.042\sigma^{2}\cos^{3}\left(\theta \right)+0.2\sigma^{2}\cos\left(\theta \right)\sin^{2}\left(\theta \right)\right]G_{yyy}+\\ \left[-0.2\sigma^{2}\cos^{3}\left(\theta \right)+0.526\sigma^{2}\cos\left(\theta \right)\sin^{2}\left(\theta \right)\right]G_{xxy}+\\ \left[0.2\sigma^{2}\sin^{3}\left(\theta \right)-0.274\sigma^{2}\cos^{2}\left(\theta \right)\sin\left(\theta \right)\right]G_{xyy} \end{array}$$

(E4) $$\begin{array}{l} T_{4}\left(XR_{\theta }\right)=\left[\sqrt{\frac{1}{12\pi }}\sigma \cos^{2}\left(\theta \right)-\sqrt{\frac{3}{4\pi }}\sigma \sin^{2}\left(\theta \right)\right]G_{xx}+\left[\sqrt{\frac{1}{12\pi }}\sigma \sin^{2}\left(\theta \right)-\sqrt{\frac{3}{4\pi }}\sigma \cos^{2}\left(\theta \right)\right]G_{yy}+\\ 4\sqrt{\frac{1}{\text{3π}}}\sigma \cos\left(\theta \right)\sin\left(\theta \right)G_{xy} \end{array}$$

(E5) $$\begin{array}{l} T_{5}\left(XR_{\theta }\right)=\left[0.059\sigma^{2}\cos^{3}\left(\theta \right)-0.204\sin^{2}\left(\theta \right)\right]G_{xx}+\left[0.059\sigma^{2}\sin^{2}\left(\theta \right)-0.204\cos^{2}\left(\theta \right)\right]G_{yy}+\\ \left[0.024\sigma^{2}\cos^{4}\left(\theta \right)+0.063\sigma^{2}\sin^{4}\left(\theta \right)-0.194\sigma^{3}\cos^{2}\left(\theta \right)\sin^{2}\left(\theta \right)\right]G_{xxxx}+\\ \left[0.063\sigma^{2}\cos^{4}\left(\theta \right)+0.024\sigma^{3}\sin^{4}\left(\theta \right)-0.194\sigma^{3}\cos^{2}\left(\theta \right)\sin^{2}\left(\theta \right)\right]G_{yyyy}+\\ \left[0.118\sigma^{2}\cos\left(\theta \right)\sin\left(\theta \right)\right]G_{xy}+\left[0.484\sigma^{3}\cos^{3}\left(\theta \right)\sin\left(\theta \right)-0.64\sigma^{3}\cos\left(\theta \right)\sin^{3}\left(\theta \right)\right]G_{xxxy}+\\ \left[-0.252\sigma^{2}\cos^{3}\left(\theta \right)\sin\left(\theta \right)-0.388\sigma^{3}\cos^{3}\left(\theta \right)\sin\left(\theta \right)+0.484\sigma^{3}\cos\left(\theta \right)\sin^{3}\left(\theta \right)\right]G_{xyyy}+\\ \left[0.378\sigma^{2}\cos^{2}\left(\theta \right)\sin^{2}\left(\theta \right)+0.92\sigma^{3}\cos^{2}\left(\theta \right)\sin^{2}\left(\theta \right)-0.194\sigma^{3}\sin^{4}\left(\theta \right)\right]G_{xxyy} \end{array}$$

(E6) $$\begin{array}{l} T_{6}\left(XR_{\theta }\right)=\sqrt{\frac{2}{2+\pi +2\cos\phi }}\cos\theta G_{x}+\sqrt{\frac{2}{2+\pi +2\cos\phi }}\sin\theta G_{y}+\sigma \cos\frac{\phi }{2}\sqrt{\frac{2}{\left(2+\pi +2\cos\phi \right)\pi }}\left(\cos^{2}\theta -\sin^{2}\theta \right)G_{xx}+\\ 4\sigma \cos\frac{\phi }{2}\sqrt{\frac{2}{\left(2+\pi +2\cos\phi \right)\pi }}\cos\theta \sin\theta G_{xy}+\sigma \cos\frac{\phi }{2}\sqrt{\frac{2}{\left(2+\pi +2\cos\phi \right)\pi }}\left(\sin^{2}\theta -\cos^{2}\theta \right)G_{yy} \end{array}$$

## References

[1] Molleda J., Granda J.C., Usamentiaga R., Garcia D.F., Laurenson D. (2014). Optimizing steel coil production: An enhanced inspection system based on anomaly detection techniques. IEEE Ind. Appl. Mag..

[2] Liu J., Tang Z., Zhang J., Chen Q., Xu P., Liu W. (2016). Visual perception-based statistical modeling of complex grain image for product quality monitoring and supervision on assembly production line. PLoS ONE.

[3] Facco P., Masiero A., Beghi A. (2013). Advances on multivariate image analysis for product quality monitoring. J. Process Control.

[4] Zakaria N.Z.I., Maz Jamilah M., Ammar Z., Shakaff A.Y.M. (2014). A bio-inspired herbal tea flavour assessment technique. Sensors.

[5] Liu C., Yang S.X., Deng L. (2015). A comparative study for least angle regression on NIR spectra analysis to determine internal qualities of navel oranges. Exp. Syst. Appl..

[6] Liu J., Tang Z., Chen Q., Xu P., Liu W., Zhu J. (2016). Toward automated quality classification via statistical modeling of grain images for rice processing monitoring. Int. J. Comput. Intell. Syst..

[7] Yazaki A., Kim C., Chan J., Mahjoubfar A., Goda K., Watanabe M., Jalali B. (2014). Ultrafast dark-field surface inspection with hybrid-dispersion laser scanning. Appl. Phys. Lett..

[8] Dong J., Zhuang D., Huang Y., Fu J. (2009). Advances in multi-sensor data fusion: Algorithms and applications. Sensors.

[9] Zhang J., Tang Z., Liu J., Tan Z., Xu P. (2016). Recognition of flotation working conditions through froth image statistical modeling for performance monitoring. Miner. Eng..

[10] Pierre G., Alex L., Da-Yi Z., Ernest H. (2002). Optical high-precision three-dimensional vision-based quality control of manufactured parts by use of synthetic images and knowledge for image-data evaluation and interpretation. Appl. Opt..

[11] Zareiforoush H., Minaei S., Alizadeh M.R., Banakar A. (2015). Potential applications of computer vision in quality inspection of rice: A review. Food Eng. Rev..

[12] Huang S.H., Pan Y.C. (2015). Automated visual inspection in the semiconductor industry: A survey. Comput. Ind..

[13] Kumar A. (2008). Computer-vision-based fabric defect detection: A survey. IEEE Trans. Ind. Electron..

[14] Liu J., Gui W., Tang Z., Hu H., Zhu J. (2013). Machine vision based production condition classification and recognition for mineral flotation process monitoring. Int. J. Comput. Intell. Syst..

[15] Liu J., Gui W., Tang Z., Yang C., Zhu J., Li J. (2013). Recognition of the operational statuses of reagent addition using dynamic bubble size distribution in copper flotation process. Miner. Eng..

[16] Huang W., Kovacevic R. (2011). A laser-based vision system for weld quality inspection. Sensors.

[17] Fan Z., Xin Z. (2011). Classification and quality evaluation of tobacco leaves based on image processing and fuzzy comprehensive evaluation. Sensors.

[18] Lin B., Jørgensen S.B. (2011). Soft sensor design by multivariate fusion of image features and process measurements. J. Process Control.

[19] Meyer F. (1994). Topographic distance and watershed lines. Signal Process..

[20] Vincent L. (1993). Morphological grayscale reconstruction in image analysis: Applications and efficient algorithms. IEEE Trans Image Process...

[21] Li M., Li H., Zhou Z.H. (2009). Semi-supervised document retrieval. Inform. Process. Manag..

[22] Wang K.C. (2014). The feature extraction based on texture image information for emotion sensing in speech. Sensors.

[23] Liu L., Fieguth P.W. (2012). Texture classification from random features. IEEE Trans. Pattern Anal. Mach. Intell..

[24] Chan C.-H., Pang G.K. (2000). Fabric defect detection by Fourier analysis. IEEE Trans. Ind. Appl..

[25] Xian G.-M. (2010). An identification method of malignant and benign liver tumors from ultrasonography based on GLCM texture features and fuzzy SVM. Exp. Syst. Appl..

[26] Galloway M.M. (1975). Texture analysis using gray level run lengths. Comput. Graph. Image Process..

[27] Guo Z., Zhang L., Zhang D. (2010). Rotation invariant texture classification using LBP variance (LBPV) with global matching. Pattern Recognit..

[28] Chen J., Hsu C.J., Chen C.C. (2009). A self-growing hidden Markov tree for wafer map inspection. J. Process Control.

[29] Hammond D.K., Simoncelli E.P. (2008). Image modeling and denoising with orientation-adapted Gaussian scale mixtures. IEEE Trans. Image Process..

[30] Yu L., He Z., Cao Q. (2010). Gabor texture representation method for face recognition using the Gamma and generalized Gaussian models. Image Vis. Comput..

[31] Guo J., Prasetyo H., Wong K. (2014). Vehicle verification using Gabor filter magnitude with Gamma distribution modelling. IEEE Signal Process. Lett..

[32] Reyes M., Escalera S. (2012). GrabCut-based human segmentation in video sequences. Sensors.

[33] Portilla J., Strela V., Wainwright M.J., Simoncelli E.P. (2003). Image denoising using scale mixture of Gaussians in the Wavelet domain. IEEE Trans. Image Process..

[34] Do M.N., Vetterli M. (2002). Wavelet-based texture retrieval using generalized Gaussian density and Kullback-Leibler distance. IEEE Trans. Image Process..

[35] Liu J., Tang Z., Zhu J., Tan Z. (2015). Statistical modelling of spatial structures-based image classification. Control Decis..

[36] Zhang Y., Lu Z., Li J. (2010). Fabric defect classification using radial basis function network. Pattern Recognit. Lett..

[37] Bair E., Tibshirani R. (2004). Semi-supervised methods to predict patient survival from gene expression data. PLoS Biol..

[38] Igual J., Salazar A., Safont G., Vergara L. (2015). Semi-supervised Bayesian classification of materials with impact-echo signals. Sensors.

[39] Jia P., Huang T., Duan S., Ge L., Yan J., Wang L. (2016). A novel semi-supervised electronic nose Learning technique: M-training. Sensors.

[40] Yoo J., Kim H.J. (2015). Target localization in wireless sensor networks using online semi-supervised support vector regression. Sensors.

[41] Vandewalle V., Biernacki C., Celeux G., Govaert G. (2013). A predictive deviance criterion for selecting a generative model in semi-supervised classification. Comput. Stat. Data Anal..

[42] Shahshahani B.M., Landgrebe D.A. (1994). The effect of unlabeled samples in reducing the small sample size problem and mitigating the Hughes phenomenon. IEEE Trans. Geosci. Remote Sens..

[43] Lee Y.S., Cho S.B. (2014). Activity recognition with android phone using mixture-of-experts co-trained with labeled and unlabeled data. Neurocomputing.

[44] Wang M., Fu W., Hao S., Tao D., Wu X. (2016). Scalable semi-supervised learning by efficient anchor graph regularization. IEEE Trans. Know. Data Eng..

[45] Zhou Z.H., Li M. (2005). Tri-training: Exploiting unlabeled data using three classifiers. IEEE Trans. Knowl. Data Eng..

[46] Blum A., Mitchell T. Combining Labeled and unlabeled data with co-training. Proceedings of the Eleventh Annual Conference on Computational Learning Theory.

[47] Ling C.X., Du J., Zhou Z.H. (2009). When Does Co-Training Work in Real Data?.

[48] Zhou Z., Li M. (2007). Semisupervised regression with cotraining-style algorithms. IEEE Trans. Knowl. Data Eng..

[49] Xiang S., Nie F., Zhang C., Zhang C. (2009). Interactive natural image segmentation via spline regression. IEEE Trans. Image Process..

[50] Friedman J.H., Roosen C.B. (1995). An introduction to multivariate adaptive regression splines. Stat. Methods Med. Res..

[51] Geusebroek J.-M., Smeulders A.W.M. (2005). A six stimulus theory for stochastic texture. Int. J. Comput. Vis..

[52] Liu J., Tang Z., Gui W., Liu W., Xu P., Zhu J. (2016). Application of statistical modeling of image spatial structures to automated visual inspection of product quality. J. Process Control.

[53] Brown M., Wohletz K.H. (1995). Derivation of the Weibull distribution based on physical principles and its connection to the Rossin-Rammler and lognormal distributions. J. Appl. Phys..

[54] Brown W.K. (1989). A theory of sequential fragmentation and its astronomical applications. J.Astrophys. Astr..

[55] Lagarias J.C., Reeds J.A., Wright M.H., Wright P.E. (1998). Convergence properties of the Nelder–Mead simplex method in low dimensions. SIAM J. Optim..

[56] Pentland A.P. (1990). Linear shape from shading. Int. J. Comput. Vis..

[57] Fujii K., Sugi S., Ando Y. (2003). Textural properties corresponding to visual perception based on the correlation mechanism in the visual system. Psychol. Res..

[58] Freeman W.T., Adelson E.H. (1991). The design and use steerable filter. IEEE Trans. Pattern Anal. Mach. Intell..

[59] Jacob M., Unser M. (2004). Design of steerable filters for feature detection using canny-like criteria. IEEE Trans. Pattern Anal. Mach. Intell..

[60] Xiang S., Nie F., Zhang C. (2010). Semi-supervised classification via local spline regression. IEEE Trans. Pattern Anal. Mach. Intell..

[61] Yadav B.K., Jindal V.K. (2001). Monitoring milling quality of rice by image analysis. Comput. Electron. Agric..

[62] Emadzadeh B., Razavi S.M.A., Farahmandfar R. (2010). Monitoring geometric characteristics of rice during processing by image analysis system and micrometer measurement. Int. Agrophys..

[63] Brosnan T., Sun D.-W. (2002). Inspection and grading of agricultural and food products by computer vision systems—A review. Comput. Electron. Agric..

[64] Brosnan T., Sun D.-W. (2004). Improving quality inspection of food products by computer vision––A review. J. Food Eng..

[65] Kurtulmuş F., Ünal H. (2015). Discriminating rapeseed varieties using computer vision and machine learning. Exp. Syst. Appl..

[66] Majumdar S., Jayas D. (2000). Classification of cereal grains using machine vision: III. Texture models. Trans. ASAE.

[67] Cocchi M., Corbellini M., Foca G., Lucisano M., Pagani M.A., Tassi L., Ulrici A. (2005). Classification of bread wheat flours in different quality categories by a wavelet-based feature selection/classification algorithm on NIR spectra. Anal. Chim. Acta.

[68] Lee T.S. (1996). Image representation using 2D Gabor wavelets. IEEE Trans. Pattern Anal. Mach. Intell..

[69] Choudhary R., Paliwal J., Jayas D. (2008). Classification of cereal grains using wavelet, morphological, colour, and textural features of non-touching kernel images. Biosyst. Eng..

[70] Kohonen T. Improved versions of learning vector quantization. Proceedings ofthe1990 IJCNN International Joint Conference on Neural Networks.

